# Dietary Pattern and Risk of Multiple Myeloma in Two Large Prospective US Cohort Studies

**DOI:** 10.1093/jncics/pkz025

**Published:** 2019-04-27

**Authors:** Dong Hoon Lee, Teresa T Fung, Fred K Tabung, Graham A Colditz, Irene M Ghobrial, Bernard A Rosner, Edward L Giovannucci, Brenda M Birmann

**Affiliations:** 1 Department of Nutrition, Harvard T.H. Chan School of Public Health, Boston, MA; 2 Department of Nutrition, Simmons University, Boston, MA; 3 Division of Medical Oncology, Department of Internal Medicine, The Ohio State University College of Medicine, Columbus, OH; 4 Department of Public Health Sciences, Washington University School of Medicine, St. Louis, MO; 5 Dana-Farber Cancer Institute, Harvard Medical School, Boston, MA; 6 Channing Division of Network Medicine, Department of Medicine, Brigham and Women’s Hospital and Harvard Medical School, Boston, MA; 7 Department of Epidemiology, Harvard T.H. Chan School of Public Health, Boston, MA

## Abstract

**Background:**

The limited data on specific dietary components and risk of multiple myeloma (MM) show no consistent association. Studies have not examined the association of dietary pattern with MM risk.

**Methods:**

In prospective cohorts of 69 751 women (Nurses’ Health Study, 1984–2014) and 47 232 men (Health Professionals Follow-up Study, 1986–2014), we examined the association between dietary pattern and risk of MM using Cox proportional hazard models. Diet was assessed repeatedly every 4 years with food frequency questionnaires and was used to calculate dietary patterns including the Alternate Healthy Eating Index-2010, Alternate Mediterranean Diet, Dietary Approaches to Stop Hypertension, Prudent and Western patterns, the empirical dietary inflammatory pattern (EDIP), and empirical dietary indices for insulin resistance (EDIR) and hyperinsulinemia (EDIH).

**Results:**

During 2 792 257 person-years of follow-up, we identified 478 incident MM cases (215 women, 263 men). In men, high EDIP was statistically significantly associated with a 16% increase in MM risk (hazard ratio [HR] = 1.16, 95% confidence interval [CI] = 1.02 to 1.32 per 1-SD increase). Moreover, EDIR and EDIH had a suggestive positive association (EDIR: HR = 1.09, 95% CI = 0.96 to 1.24; and EDIH: HR = 1.11, 95% CI = 0.97 to 1.28 per 1-SD increase). We observed no other associations with MM risk in men and no associations for any dietary pattern with MM risk in women.

**Conclusions:**

We present the first evidence for a role of diets with higher inflammatory or insulinemic potential in MM development. Further studies are warranted to explore these associations in other populations, including the apparent restriction to men.

Multiple myeloma (MM) is a plasma cell neoplasm that was expected to account for more than 30 700 new cancer diagnoses and more than 12 700 deaths in the United States in 2018 (1). Although MM survival has improved because of the development of novel treatments, relative 5- and 10-year survival rates remain as low as 51.6% and 26.8%, respectively (2–7). Further, current understanding of MM etiology is insufficient to inform prevention strategies; most established risk factors for MM are not modifiable (8–10).

In the pathogenesis of MM, upregulation of inflammatory pathways that mediate nuclear factor-kB and interleukin-6, as well as dysregulation of endogenous growth factors including insulin-like growth factor-1 and insulin, have well-documented roles (11–17). Thus, modifiable factors that modulate these pathways may also influence MM risk. Obesity, which is characterized by upregulated inflammation and deregulated endogenous growth factors (18,19), has been consistently positively associated with MM risk (20–22). In fact, obesity is considered the first and only modifiable risk factor for MM (23) .

Diet may influence MM risk by modulating the aforementioned pathways (24,25). The literature on diet and MM risk is limited but suggests greater consumption of fish and cruciferous vegetables and less consumption of desserts and sweets may lower risk (26–28). To our knowledge, no studies have yet examined dietary patterns, rather than intake of individual foods or nutrients, in relation to MM risk. The study of dietary pattern as a risk factor has advantages over the study of individual foods or nutrients because dietary pattern accounts for additive, interactive, or synergistic effects of multiple foods and nutrients and has more intuitive implications for clinical and public health recommendations (29–32).

Dietary pattern can be characterized using a variety of methods. One approach uses a priori-defined scores based on foods or nutrients that reflect adherence to a dietary recommendation or characteristic diet, such as the Alternative Healthy Eating Index-2010 (AHEI-2010), Alternative Mediterranean Diet (aMED), and Dietary Approach to Stop Hypertension (DASH) (33–35). Another approach derives a posteriori-defined dietary patterns, such as Prudent and Western dietary patterns, using statistical exploratory methods like principal component analysis (36,37). An additional approach uses empirically derived dietary indices, including Empirical Dietary Inflammatory Pattern (EDIP), Empirical Dietary Index for Insulin Resistance (EDIR), and Empirical Dietary Index for Hyperinsulinemia (EDIH) (38,39). These dietary indices have shown robust associations with inflammatory and insulin response biomarkers and allow for assessment of the inflammatory (38) or insulinemic potential (39) of an individual’s diet.

We conducted the current study to examine the association between a variety of dietary patterns and risk of MM in two large prospective US cohorts with up to 30 years of follow-up and repeated assessment of usual adult dietary habits.

## Methods

### Study Population

The Nurses’ Health Study (NHS) began in 1976 when 121 700 female registered nurses aged 30 to 55 years completed the enrollment questionnaire (40). The Health Professionals Follow-up Study (HPFS) began in 1986 when 51 529 male health professionals aged 40 to 75 years returned the initial questionnaire (41). Participants in both cohorts completed biennial follow-up questionnaires to update information on lifestyle and medical history, with follow-up rates that exceed 90%. The study protocol was approved by the institutional review boards of the Brigham and Women’s Hospital and Harvard T.H. Chan School of Public Health, and those of participating registries as required.

### Dietary Assessment

Diet was assessed by a validated, semiquantitative food frequency questionnaire (FFQ) every 4 years beginning in 1980 (NHS) and 1986 (HPFS) (42–44). Each FFQ contained approximately 130 items (except for the 1980 FFQ; 61 items), for which standard portion sizes with nine frequency choices ranging from less than one time per month to six or more times per day were provided. For this study, we used the data from 1984 (NHS) and 1986 (HPFS) onward because the expanded number of FFQ items was important to characterize dietary patterns. All FFQs returned by participants were uses to compute the dietary patterns described in Table 1.

**Table 1.** Description of dietary patterns*

Type	Principle	Calculation
A priori-defined scores		
AHEI-2010	Based on 11 dietary components shown to be associated with lower risk of chronic disease (33)	Emphasizes higher consumption of vegetables, fruits, whole grains, nuts and legumes, long-chain omega-3 fatty acids, and polyunsaturated fatty acids and lower consumption of sugar-sweetened beverages, red and processed meat, sodium, trans fat, and moderate alcohol, as captured by the FFQ. Each component scored from 0 to 10 points based on predefined criteria.
		Total score ranged from 0 to 110 points, with a higher score considered to represent a healthier diet.
aMED	Based on foods and nutrients that reflect a typical Mediterranean diet (35)	Awards 1 point per item consumed at a level above cohort-specific median for vegetables, legumes, fruits, nuts, whole grains, fish, and monounsaturated fat-to-saturated fat ratio and for intake below cohort-specific median for red/processed meats. For alcohol intake, score awards 1 point if intake 5–15 g/d for women and 10–25 g/d for men.
		Total score ranged from 0 to 9 points; higher score represents closer adherence to Mediterranean (and favorable) diet.
DASH	Based on foods and nutrients that are recommended based on DASH trial, which identified a dietary pattern associated with reduced risk of hypertension (34)	Awards points for high intake of fruits, vegetables, nuts and legumes, low-fat dairy products, and whole grains, and for low intake of red/processed meats, sweets, and sodium. For each component, participants in the lowest cohort-specific quintile received 1 point and those in the highest quintile received 5 points for consumption of healthy food or nutrient; scoring was reversed for unhealthy foods or nutrients.
		Total score ranged 8–40 points; higher scores considered more favorable to hypertension prevention.
A posteriori-defined dietary patterns		
PrudentWestern	Based on FFQs (approximately 40 food groups) using principle component analysis with orthogonal transformation (36, 37)As above	Consultation of eigenvalues and scree plot identified 2 major dietary patterns to retain for analysis: Prudent pattern (high intake of vegetables, fruits, legumes, whole grains, and fish) and Western pattern (high intake of red/processed meats, high-fat dairy products, refined grains, and sweets/desserts).
		Individual scores calculated for each pattern based on reported food intakes and corresponding factor loadings of the foods.

Empirically derived dietary indices		
EDIP	Based on 39 predefined food groups from FFQs (38, 39)As aboveAs above	EDIP was derived using reduced rank regression and stepwise linear regression to identify food groups most predictive of 3 inflammatory markers (interleukin-6, C-reactive protein, and tumor necrosis factor-alpha receptor). Nine food groups were positively associated (processed and red meat, refined grains, high-energy beverages, etc.), and 9 food groups were inversely associated (coffee, wine, leafy green vegetables, etc.).

EDIREDIH		
		EDIR was derived using stepwise linear regression to identify food groups most predictive of hyperinsulinemia (C-peptide). Ten food groups were positively associated (processed and red meat, non-fatty fish, margarine, creamy soup, etc.), and 8 food groups were inversely associated (wine, beer, dark yellow vegetables, etc.).
		EDIH was derived using stepwise linear regression to identify food groups most predictive of a surrogate of insulin resistance (triglyceride to HDL cholesterol ratio). Thirteen food groups were positively associated (processed and red meat, poultry, non-fatty fish, creamy soup, french fries, etc.), and 5 food groups were inversely associated (wine, green leafy vegetables, high-fat dairy, etc.).
		Food groups identified as most strongly predicting circulating levels of corresponding biomarkers were weighted by regression coefficients obtained from final stepwise linear regression model and then summed to create the score. More positive scores indicate a more inflammatory or insulinemic (ie, associated with hyperinsulinemia or insulin resistance) diet; more negative scores indicate a less inflammatory or insulinemic diet. A summary of the identified food groups is provided in Supplementary Table 3 (available online).

*AHEI = alternate healthy eating index-2010; aMED = alternate Mediterranean diet; DASH = dietary approaches to stop hypertension; EDIH = empirical dietary index for hyperinsulinemia; EDIP = empirical dietary inflammatory pattern; EDIR = empirical dietary index for insulin resistance; FFQ = food frequency questionnaire; HDL = high-density lipoprotein.

### Covariate Assessment

Adult height and current weight were reported on the enrollment questionnaires, with weight updated biennially. Body mass index (BMI) was calculated as weight divided by height squared (kg/m^2^). Participants reported information on other covariates such as physical activity and aspirin use from the biennial questionnaires.

### Case Identification

We identified most new diagnoses of cancer by self-report on the biennial questionnaires. A small number of cases were ascertained via death follow-up. Deaths were identified by next-of-kin, postal service, or routine searches of the National Death Index (45,46). For each newly identified case of MM, we sought permission to obtain the medical records, which were reviewed by a physician or trained reviewer blinded to exposure status to confirm the diagnosis and diagnosis date. When medical records were not available, we sought to confirm the diagnosis via linkage to applicable state tumor registries.

### Statistical Analysis

Among those who returned FFQs in 1984 (NHS, n = 81 599) and 1986 (HPFS, n = 49 197), we excluded participants with a baseline history of cancer (n = 4450 women, n = 1965 men) or implausible total energy intake (n = 7398 women with <500 or >3500 kcal/d; n = 0 men with <800 or >4200 kcal/d), leaving 69 751 women and 47 232 men in the analyses.

Person-time was calculated from baseline (1984 for NHS, 1986 for HPFS) until the earliest among dates of MM or other cancer (except non-melanoma skin cancer) diagnosis, death, or the end of follow-up (June 2014 for NHS, January 2014 for HPFS). We used cumulative averages of the respective dietary pattern (updated with repeated measures through the diagnosis or censoring time) to characterize long-term dietary pattern and reduce measurement error (47). Because tests for nonlinearity using polynomial terms of dietary patterns were not statistically significant, and because the patterns did not have intuitive a priori categories, we used each dietary pattern as a continuous variable and modeled risk per 1-SD increase. Cox proportional hazards regression models stratified on age and questionnaire cycle were used to compute hazard ratios (HRs) and 95% confidence intervals (CIs) for MM risk associated with the individual dietary patterns. In multivariable models, we adjusted for potential confounders including cumulative averages of total energy intake (continuous) and BMI (continuous) (22). We considered as potential covariables but ultimately did not include physical activity (48) and regular aspirin use (49) in the final models because their addition changed the estimates by less than 1% for most models.

The primary analyses were conducted separately by cohort (sex); for secondary analyses we pooled the data and included sex as an additional stratification variable. We performed Wald tests for interaction by including cross-product terms for the given dietary pattern and sex. We conducted subgroup analyses to explore whether the associations between respective dietary patterns and MM risk differed by BMI (<25 or ≥25 kg/m^2^) and tested for potential interaction by adding the cross-product term for dietary pattern and BMI to the models. We further examined the association of cross-classified dietary pattern (tertile) and BMI (binary) with MM risk. Lastly, we conducted sensitivity analyses using tertile of dietary patterns as well as baseline and recent (closest measures before the diagnosis or censoring time) dietary patterns.

All statistical analyses were performed using SAS 9.4 (SAS Institute) and assumed a two-tailed alpha error of 0.05.

## Results

We confirmed incident MM in 215 women (NHS) and 263 men (HPFS) over 30 years of follow-up (1 709 737 person-years in NHS; 1 082 520 person-years in HPFS). The mean age was slightly younger for women (50.7 years) than men (54.4 years). The mean BMI was similar (approximately 25–26 kg/m^2^) for women and men (Table 2). Participants with a higher AHEI-2010, aMED, DASH, and Prudent pattern tended to have lower BMI; higher physical activity; lower intakes of red and processed meat, refined carbohydrates, and sugar-sweetened beverage; and higher intakes of whole grains, fruits, and vegetables (Supplementary Table 1, available online). Western pattern, EDIP, EDIR, and EDIH showed opposite trends in general, with no marked differences by sex.

**Table 2.** Age-standardized baseline characteristics of participants*

Characteristic	NHS (69 751 women)	HPFS (47 232 men)
Age, y†	50.7 (7.1)	54.4 (9.7)
BMI, kg/m^2^	25.0 (4.6)	25.6 (3.3)
Physical activity, MET-h/wk	12.7 (15.2)	20.9 (29.1)
Regular aspirin use, % ‡	40.1	20.0
Diet intake		
Calorie intake, kcal/d	1749 (523)	1989 (619)
Alcohol, g/d	6.9 (11.2)	11.3 (15.4)
Processed meat, servings/wk	2.2 (2.3)	2.6 (3.0)
Red meat, servings/wk	4.5 (2.8)	4.3 (3.2)
Poultry, servings/wk	2.1 (1.7)	2.5 (2.0)
Fish, servings/wk	2.2 (1.8)	2.9 (2.3)
Whole grain, servings/wk	6.5 (7.1)	10.0 (9.7)
Refined carbohydrates, servings/wk	8.4 (7.1)	8.6 (7.5)
Fruits, servings/wk	9.9 (7.4)	11.3 (9.1)
Vegetables, servings/wk	16.6 (10.5)	21.7 (15.2)
High-fat dairy, servings/wk	7.6 (7.3)	6.8 (7.2)
Low-fat dairy, servings/wk	6.4 (6.8)	7.0 (7.5)
Nuts, servings/wk	2.2 (3.3)	3.4 (4.8)
Coffee, servings/wk	17.5 (13.6)	29.3 (20.5)
SSB, servings/wk	2.1 (4.1)	2.5 (4.2)

* Data were presented as mean (SD) (unless otherwise specified). BMI = body mass index; HPFS = Health Professionals Follow-Up Study; MET = metabolic equivalent task; NHS = Nurses’ Health Study; SSB = sugar-sweetened beverage.

† Age was not standardized.

‡ Updated information over the follow-up due to no baseline information in men.

Moderate to high correlations were evident among AHEI-2010, aMED, DASH, and Prudent patterns (Spearman correlation = 0.51 to 0.80) (Supplementary Table 2, available online). These patterns were inversely correlated with the Western pattern, EDIP, and EDIH. Further, there were moderate to high correlations among three empirical dietary indices (Spearman correlation = 0.57 to 0.76).

In the cohort (sex)-specific analyses of dietary pattern and MM risk, we observed a statistically significant positive association for cumulative average EDIP in men (Table 3). After adjusting for age, energy intake, and BMI, each 1-SD increase of cumulative average EDIP was associated with a 16% increased risk of MM (HR = 1.16, 95% CI = 1.02 to 1.32). EDIR and EDIH had suggestive positive associations with MM risk in men in the fully adjusted models (EDIR: HR per 1-SD increase = 1.09, 95% CI = 0.96 to 1.24; EDIH: HR per 1-SD increase = 1.11, 95% CI = 0.97 to 1.28). Other dietary patterns were not statistically significantly associated with MM risk in men. In women, none of the dietary patterns had statistically significant associations with MM risk (Table 3).

**Table 3.** Association between cumulative average dietary pattern and multiple myeloma risk in women and men

	HR (95% CI) per 1-SD increase*,†	
Dietary pattern	Women	Men
AHEI-2010		
Age- and energy-adjusted‡	1.02 (0.89 to 1.18)	1.03 (0.91 to 1.17)
Multivariable-adjusted§	1.04 (0.90 to 1.20)	1.05 (0.93 to 1.20)
aMED		
Age- and energy-adjusted‡	0.97 (0.83 to 1.13)	0.95 (0.83 to 1.08)
Multivariable-adjusted§	0.99 (0.84 to 1.15)	0.96 (0.84 to 1.10)
DASH		
Age- and energy-adjusted‡	0.98 (0.85 to 1.14)	0.93 (0.82 to 1.06)
Multivariable-adjusted§	1.00 (0.86 to 1.16)	0.95 (0.83 to 1.08)
Prudent		
Age- and energy-adjusted‡	0.95 (0.80 to 1.11)	0.95 (0.83 to 1.10)
Multivariable-adjusted§	0.95 (0.80 to 1.11)	0.96 (0.84 to 1.11)
Western		
Age- and energy-adjusted‡	1.06 (0.87 to 1.30)	1.05 (0.89 to 1.24)
Multivariable-adjusted§	1.03 (0.84 to 1.26)	1.02 (0.86 to 1.21)
EDIP		
Age- and energy-adjusted‡	1.06 (0.91 to 1.23)	1.18 (1.03 to 1.35)
Multivariable-adjusted§	1.01 (0.87 to 1.18)	1.16 (1.02 to 1.32)
EDIR		
Age- and energy-adjusted‡	1.00 (0.86 to 1.17)	1.11 (0.98 to 1.27)
Multivariable-adjusted§	0.95 (0.80 to 1.11)	1.09 (0.96 to 1.24)
EDIH		
Age- and energy-adjusted‡	1.08 (0.91 to 1.28)	1.14 (0.99 to 1.31)
Multivariable-adjusted§	1.02 (0.86 to 1.22)	1.11 (0.97 to 1.28)

* Sex-specific SD was used. AHEI = alternate healthy eating index-2010; aMED = alternate Mediterranean diet; BMI = body mass index; CI = confidence interval; DASH = dietary approaches to stop hypertension; EDIH = empirical dietary index for hyperinsulinemia; EDIP = empirical dietary inflammatory pattern; EDIR = empirical dietary index for insulin resistance; HR = hazard ratio.

† Case per person-years: 215 per 1 709 737 for women; 263 per 1 082 520 for men.

‡ Adjusted for age in years and cumulative average energy intake (continuous).

§ Additionally adjusted for cumulative average BMI (continuous).

When we examined the association of cross-classified dietary patterns and BMI in relation to MM risk, we found statistically significant associations of EDIP and EDIH cross-classified with BMI in men (Table 5). Compared with lean men in the lowest (eg, “healthiest”) tertile of EDIP and EDIH, overweight or obese men in the highest (eg, “least healthy”) tertile had a 96% and 74% increased risk of MM, respectively (EDIP: HR = 1.96, 95% CI = 1.22 to 3.13; EDIH: HR = 1.74, 95% CI = 1.10 to 2.75). We found no other statistically significant association for BMI cross-classified with dietary patterns for men or women (Tables 4 and 5). When we stratified the analyses by BMI, the association between dietary pattern and MM risk did not differ statistically significantly by BMI in men, although EDIP, EDIR, and EDIH tended to show stronger positive associations with MM risk for lean than for heavier men (Supplementary Table 4, available online).

**Table 4.** Association of cross-classified cumulative average dietary pattern and BMI with multiple myeloma risk in women

	Healthiest	Medium	Unhealthiest
Presumed healthy dietary patterns*	Tertile 3	Tertile 2	Tertile 1
AHEI-2010			
BMI<25			
Cases	34	38	24
HR (95% CI)	1 (reference)*	1.34 (0.84 to 2.14)	0.96 (0.56 to 1.62)
BMI≥25			
Cases	39	40	40
HR (95% CI)	1.36 (0.86 to 2.16)	1.27 (0.80 to 2.01)	1.35 (0.85 to 2.14)
aMED			
BMI<25			
Cases	32	38	26
HR (95% CI)	1 (reference)*	1.21 (0.75 to 1.95)	0.94 (0.55 to 1.61)
BMI≥25			
Cases	40	37	42
HR (95% CI)	1.38 (0.86 to 2.20)	1.14 (0.71 to 1.85)	1.30 (0.81 to 2.11)
DASH			
BMI<25			
Cases	33	35	28
HR (95% CI)	1 (reference)*	1.22 (0.76 to 1.98)	1.11 (0.66 to 1.85)
BMI≥25			
Cases	44	35	40
HR (95% CI)	1.47 (0.93 to 2.31)	1.12 (0.69 to 1.81)	1.43 (0.89 to 2.28)
Prudent			
BMI<25			
Cases	30	31	35
HR (95% CI)	1 (reference)*	1.07 (0.64 to 1.79)	1.21 (0.72 to 2.02)
BMI≥25			
Cases	42	38	39
HR (95% CI)	1.33 (0.83 to 2.14)	1.21 (0.74 to 1.98)	1.41 (0.85 to 2.33)

Presumed unhealthy dietary patterns*	Tertile 1	Tertile 2	Tertile 3

Western			
BMI<25			
Cases	37	38	21
HR (95% CI)	1 (reference)*	1.29 (0.81 to 2.06)	0.91 (0.50 to 1.66)
BMI≥25			
Cases	34	47	38
HR (95% CI)	1.05 (0.66 to 1.68)	1.51 (0.97 to 2.36)	1.34 (0.79 to 2.26)
EDIP			
BMI<25			
Cases	28	37	31
HR (95% CI)	1 (reference)*	1.56 (0.95 to 2.56)	1.89 (1.13 to 3.16)
BMI≥25			
Cases	36	37	46
HR (95% CI)	2.00 (1.21 to 3.28)	1.50 (0.92 to 2.47)	1.67 (1.04 to 2.69)
EDIR			
BMI<25			
Cases	34	43	19
HR (95% CI)	1 (reference)*	1.60 (1.01 to 2.52)	1.06 (0.59 to 1.90)
BMI≥25			
Cases	38	36	45
HR (95% CI)	1.74 (1.09 to 2.78)	1.29 (0.80 to 2.07)	1.43 (0.90 to 2.27)
EDIH			
BMI<25			
Cases	34	36	26
HR (95% CI)	1 (reference)*	1.46 (0.90 to 2.36)	1.62 (0.94 to 2.78)
BMI≥25			
Cases	38	42	39
HR (95% CI)	1.75 (1.10 to 2.78)	1.52 (0.96 to 2.40)	1.41 (0.87 to 2.30)

* All models adjusted for age in years and cumulative average energy intake (continuous). Reference group is those with low BMI and the healthiest dietary pattern. For the presumed healthy dietary patterns in the top half of the table, the reference group includes participants with a low BMI and a dietary pattern score in the highest tertile (eg, greatest adherence to the healthy dietary pattern), whereas the highest-risk group includes participants with a higher BMI and a dietary pattern score in the lowest tertile (eg, least adherence to the healthier dietary pattern). For the presumed unhealthy dietary patterns in the lower half of the table, the reference group includes participants with a low BMI and a dietary pattern score in the lowest tertile (eg, least adherence to the less healthy dietary pattern), whereas the highest-risk group includes participants with a higher BMI and a dietary pattern score in the highest tertile (eg, greatest adherence to the less healthy dietary pattern). AHEI-2010 = alternate healthy eating index-2010; aMED = alternate Mediterranean diet; BMI = body mass index; CI = confidence interval; DASH = dietary approaches to stop hypertension; EDIH = empirical dietary index for hyperinsulinemia; EDIP = empirical dietary inflammatory pattern; EDIR = empirical dietary index for insulin resistance; HR = hazard ratio.

**Table 5.** Association of cross-classified cumulative average dietary pattern and BMI with multiple myeloma risk in men

	Healthiest	Medium	Unhealthiest
Presumed healthy dietary patterns*	Tertile 3	Tertile 2	Tertile 1
AHEI-2010			
BMI <25			
Cases	39	36	25
HR (95% CI)	1 (reference)*	1.21 (0.76 to 1.91)	1.00 (0.60 to 1.66)
BMI ≥25			
Cases	60	55	48
HR (95% CI)	1.55 (1.03 to 2.33)	1.27 (0.84 to 1.93)	1.12 (0.73 to 1.72)
aMED			
BMI <25			
Cases	37	32	31
HR (95% CI)	1 (reference)*	0.98 (0.60 to 1.58)	1.27 (0.78 to 2.08)
BMI ≥25			
Cases	52	58	53
HR (95% CI)	1.31 (0.86 to 2.01)	1.29 (0.85 to 1.96)	1.31 (0.85 to 2.02)
DASH			
BMI <25			
Cases	36	40	24
HR (95% CI)	1 (reference)*	1.64 (1.04 to 2.59)	1.24 (0.73 to 2.09)
BMI≥25			
Cases	52	62	49
HR (95% CI)	1.47 (0.95 to 2.25)	1.67 (1.10 to 2.54)	1.47 (0.94 to 2.29)
Prudent			
BMI<25			
Cases	39	35	26
HR (95% CI)	1 (reference)*	1.10 (0.69 to 1.76)	0.98 (0.58 to 1.64)
BMI≥25			
Cases	61	55	47
HR (95% CI)	1.35 (0.90 to 2.03)	1.25 (0.82 to 1.91)	1.16 (0.74 to 1.83)

Presumed unhealthy dietary patterns*	Tertile 1	Tertile 2	Tertile 3

Western			
BMI <25			
Cases	38	28	34
HR (95% CI)	1 (reference)*	1.34 (0.80 to 2.23)	1.54 (1.02 to 2.32)
BMI ≥25			
Cases	47	65	51
HR (95% CI)	0.97 (0.59 to 1.60)	1.29 (0.84 to 1.99)	1.16 (0.72 to 1.86)
EDIP			
BMI <25			
Cases	24	40	36
HR (95% CI)	1 (reference)*	1.71 (1.01 to 2.88)	1.62 (0.99 to 2.65)
BMI ≥25			
Cases	46	49	68
HR (95% CI)	1.55 (0.93 to 2.58)	1.55 (0.94 to 2.56)	1.96 (1.22 to 3.13)
EDIR			
BMI <25			
Cases	33	37	30
HR (95% CI)	1 (reference)*	1.06 (0.64 to 1.76)	1.42 (0.92 to 2.19)
BMI ≥25			
Cases	46	57	60
HR (95% CI)	1.06 (0.66 to 1.71)	1.17 (0.75 to 1.84)	1.24 (0.80 to 1.93)
EDIH			
BMI <25			
Cases	32	34	34
HR (95% CI)	1 (reference)*	1.83 (1.10 to 3.05)	1.62 (1.04 to 2.52)
BMI ≥25			
Cases	48	56	59
HR (95% CI)	1.38 (0.84 to 2.25)	1.53 (0.97 to 2.40)	1.74 (1.10 to 2.75)

* All models adjusted for age in years and cumulative average energy intake (continuous). Reference group is those with low BMI and the healthiest dietary pattern. For the presumed healthy dietary patterns in the top half of the table, the reference group includes participants with a low BMI and a dietary pattern score in the highest tertile (eg, greatest adherence to the healthy dietary pattern), whereas the highest-risk group includes participants with a higher BMI and a dietary pattern score in the lowest tertile (eg, least adherence to the healthier dietary pattern). For the presumed unhealthy dietary patterns in the lower half of the table, the reference group includes participants with a low BMI and a dietary pattern score in the lowest tertile (eg, least adherence to the less healthy dietary pattern), whereas the highest-risk group includes participants with a higher BMI and a dietary pattern score in the highest tertile (eg, greatest adherence to the less healthy dietary pattern). AHEI-2010 = alternate healthy eating index-2010; aMED = alternate Mediterranean diet; BMI = body mass index; CI = confidence interval; DASH = dietary approaches to stop hypertension; EDIH = empirical dietary index for hyperinsulinemia; EDIP = empirical dietary inflammatory pattern; EDIR = empirical dietary index for insulin resistance; HR = hazard ratio.

In the sensitivity analyses using baseline and recent dietary pattern, the baseline EDIP, EDIR, and EDIH showed statistically significant positive associations with MM risk for men but no associations for women (Supplementary Table 5, available online). In contrast, recent dietary patterns were not associated with MM risk in women or men (Supplementary Table 6, available online). Analyses of tertile of dietary patterns showed results consistent with those already described (Supplementary Tables 7 and 8, available online). In the analyses of pooled data, we found no association between cumulative average dietary patterns and MM risk overall (Supplementary Table 9, available online) or when jointly classified with BMI (Supplementary Table 10, available online). However, 1-SD increases of EDIP, EDIR, and EDIH were statistically significantly associated with an 18–29% increased risk of MM in lean participants in the pooled analyses stratified by BMI (Supplementary Table 11, available online).

## Discussion

In two large US prospective cohorts, we observed a statistically significantly positive association between EDIP, a dietary index associated with pro-inflammatory biomarkers, and MM risk in men. EDIR and EDIH, dietary indices associated positively with insulin-related biomarkers, showed suggestive positive associations with MM risk in men. Other dietary patterns were not statistically significantly associated with MM in men, and we did not find any statistically significant association of dietary pattern with MM risk in women.

To our knowledge, no studies to date have examined the association between dietary pattern and MM risk. Further, relatively few published studies have examined the association of individual foods or nutrients with MM risk. Of those, several case-control studies have reported that higher intake of vegetables (50,51), especially cruciferous vegetables (26,27), and fish (26–28,50,52) were associated with a lower risk of MM, whereas higher intake of some dairy products were positively associated with higher risk of MM (26). Overall, current knowledge on the association between diet and MM risk is limited and inconclusive. Moreover, the previous studies had relatively small sample size and low response rates. Also, recall bias cannot be ruled out for case-control studies; recall of diet may differ by disease status, because patients generally have cultivated a sense of which foods are healthy or unhealthy.

In the present study, we evaluated the association of diet with MM risk using dietary pattern rather than individual foods or nutrients. Our finding of a positive association of the EDIP in men is consistent with our hypothesis that immune-modulating or pro-inflammatory factors influence MM risk (13,53,54). Of interest, dysregulation of endogenous growth factors such as insulin-like growth factor-1 and insulin also contributes to MM pathogenesis (12,15,16,55), lending plausibility to our observation of suggestive positive associations with MM risk for EDIR and EDIH, which were derived to characterize the insulinemic potential of diet, particularly related to insulin resistance and hyperinsulinemia, respectively (39). The latter associations also had apparent restrictions to men.

Interestingly, we observed statistically significant and plausible associations of cross-classifications of EDIP and obesity, and of EDIH and obesity, with MM risk, also predominantly in men. Obesity is the only established modifiable risk factor for MM (23,56). Although we had limited power for those analyses, we consistently observed that individuals with the greatest dietary potential for heightened inflammation or hyperinsulinemia and also higher BMI had the greatest increase in MM risk. Higher adiposity is associated with higher levels of pro-inflammatory cytokines and endogenous growth factors and with lower levels of circulating adiponectin. It is biologically plausible that low dietary quality in the setting of greater adiposity has synergistic adverse effects on MM pathogenesis. Another interesting observation was that stratified analyses generally showed a stronger positive association between inflammatory or insulinemic diets and MM risk in lean individuals compared with overweight or obese individuals. Although the interactions between these diet patterns and BMI were mostly not statistically significant, these findings suggest that inflammatory or insulinemic diets may be a stronger risk factor of MM in individuals with lower adiposity.

The other dietary patterns that we examined in this study did not have a clear association with MM risk in men or in women. Previous studies have shown an inverse association of AHEI-2010, aMED, DASH, and Prudent patterns, as well as a positive association of Western pattern, with major chronic diseases and certain solid tumors (32,33,57–61). Further, these dietary patterns were shown to be associated with inflammation (24,62). Thus, our null findings were somewhat unexpected and inconsistent with those for other chronic diseases. Reassuringly, our findings for AHEI-2010, aMED, and DASH did not suggest an increased risk of MM, supporting that adherence to current recommendations of dietary patterns based on scientific evidence for the prevention of chronic diseases does not confer harm from MM risk. The reasons for the discrepancy of findings for the dietary pattern scores and for EDIP, EDIR, and EDIH are not immediately clear. The latter dietary indices were directly derived from food groups that are most predictive of biomarkers of inflammation, insulin resistance, and hyperinsulinemia, respectively, and thus may more directly or more precisely capture the immune-modulating aspects of diet that are most relevant to MM development. Alternatively, more limited variability in the other dietary score variables, and possibly residual confounding by unknown or unmeasured risk factors, may have hindered detection of a subtler association of other dietary patterns with MM risk.

Unlike in men, dietary patterns did not show any association or trend in relation to MM risk in women, except that we found a statistically significantly positive association of EDIP and EDIH with MM risk among lean women. The observed sex difference may be due to chance or to uncontrolled confounding by as-yet-unknown risk factors for MM that vary by sex. Alternatively, the findings may reflect true sex differences in physiologic effects of dietary pattern on the development of MM. In our cohorts, we have seen sex differences for other risk factors for MM. For example, both young adult and later adult BMI were stronger risk factors for men compared with women (22). Furthermore, regular aspirin intake showed stronger inverse association with MM risk in men than in women (49). Overall, the literature is variable with regard to reports of sex differences in these factors and does not offer clarification of our present findings. However, we note that MM incidence is generally 40–50% higher in men than in women (7). With the present sample sizes, we had limited statistical power to detect statistically significant interactions of dietary pattern and sex. Our secondary analyses pooling the data for women and men did not replicate the statistically significant findings in men. The present observation of sex differences for the dietary index associations with MM risk requires further exploration in larger study populations.

This study has considerable strengths. Detailed diet data allowed us to compute and compare a variety of dietary patterns to examine their association with MM risk. Further, repeated measures of dietary pattern were available over a long follow-up period, permitting an examination of the influence of timing of “exposure.” In fact, the associations for EDIP, EDIR, and EDIH appeared somewhat stronger for baseline measures than for the cumulative averages, whereas the associations were attenuated when based only on the most recent dietary assessment. The latter observation may reflect an influence of subclinical disease on dietary habits, though the most recent dietary assessment tended, on average, to be approximately 2 years before diagnosis of MM. The findings may also suggest an earlier critical period for an influence of dietary pattern on MM development, although we did not have sufficient statistical power to stratify the analyses by follow-up interval to elucidate this further. Other strengths include the prospective design and the homogeneity of the study population that makes residual confounding by race/ethnicity unlikely. Several limitations also warrant attention. First, measurement error in assessing dietary pattern is inevitable. However, use of cumulative average of repeated measures may reduce the measurement error of long-term intake. Given the prospective study design, the measurement error is likely nondifferential. Second, although we adjusted for some known and potential confounders, we cannot rule out residual confounding by unknown or unmeasured factors such as family history of lymphoid malignancy (9,10). Lastly, our study population consisted of predominantly white health professionals, which may limit the generalizability of the findings if dietary pattern is correlated with aspects of race or ethnicity that also underlie its association with MM.

In conclusion, our findings show evidence that diets with inflammatory or insulinemic potential may play a role in MM development, with apparent restrictions to men. Our study warrants confirmation in populations with larger sample size and greater racial and ethnic diversity as well as in relation to risk of progression from monoclonal gammopathy of undetermined significance to MM.

## Funding

This work was supported by the National Institutes of Health (UM1 CA167552, R01 HL35464, UM1 CA186107, P01 CA87969, K99 CA207736, R00 CA207736, and R21 CA198239).

## Notes

Affiliations of authors: Department of Nutrition, Harvard T.H. Chan School of Public Health, Boston, MA (DHL, TTF, FKT, ELG); Department of Nutrition, Simmons University, Boston, MA (TTF); Division of Medical Oncology, Department of Internal Medicine, The Ohio State University College of Medicine, Columbus, OH (FKT); Department of Public Health Sciences, Washington University School of Medicine, St. Louis, MO (GAC); Dana-Farber Cancer Institute, Harvard Medical School, Boston, MA (IMG); Channing Division of Network Medicine, Department of Medicine, Brigham and Women’s Hospital and Harvard Medical School, Boston, MA (BAR, ELG, BMB); Department of Epidemiology, Harvard T.H. Chan School of Public Health, Boston, MA (ELG).

We would like to thank the following state cancer registries for their help: AL, AZ, AR, CA, CO, CT, DE, FL, GA, ID, IL, IN, IA, KY, LA, ME, MD, MA, MI, NE, NH, NJ, NY, NC, ND, OH, OK, OR, PA, RI, SC, TN, TX, VA, WA, WY. The authors assume full responsibility for analyses and interpretation of these data.

The authors declared no conflicts of interest.

## Supplementary Material

Supplementary_Material_pkz025
