# Supplementary material for: Dietary Pattern and Risk of Multiple Myeloma in Two Large Prospective US Cohort Studies
Source: JNCI Cancer Spectr. 2019 Apr 27;3(2):pkz025. doi: 10.1093/jncics/pkz025 (PMC6532330; doi:10.1093/jncics/pkz025)
Supplement: Supplementary_Material_pkz025 [file supplementary_material_pkz025.docx]

**Supplementary online contents**

**Table S1.** Age standardized baseline characteristics of participants according to tertile of dietary patterns

**Table S2.** Spearman correlations among dietary patterns

**Table S3.** Summary of food items associated with the empirically derived dietary patterns

**Table S4.** Association between cumulative average dietary pattern and multiple myeloma risk stratified by BMI

**Table S5.** Association between baseline dietary pattern and multiple myeloma risk in women and men

**Table S6.** Association between recent dietary pattern and multiple myeloma risk in women and men

**Table S7.** Association between tertile of cumulative average dietary pattern and multiple myeloma risk in women

**Table S8.** Association between tertile of cumulative average dietary pattern and multiple myeloma risk in men

**Table S9.** Association between cumulative average dietary pattern and multiple myelomaⱡ risk (pooled results of women and men)

**Table S10.** Association of cross-classified cumulative average dietary pattern and BMI with multiple myeloma risk (pooled results of women and men)

**Table S11.** Association between cumulative average dietary pattern and multiple myeloma risk stratified by BMI (pooled results of women and men)

This supplementary material has been provided by the authors to give readers additional information about their work.

| **Table S1. Age-standardized baseline characteristics of participants according to tertile of dietary patterns** | | | | | | |
| --- | --- | --- | --- | --- | --- | --- |
|  | **NHS (69,751 women)** | | | **HPFS (47,232 Men)** | | |
| **AHEI-2010** | **Tertile 1** | **Tertile 2** | **Tertile 3** | **Tertile 1** | **Tertile 2** | **Tertile 3** |
| Age, year^b^ | 49.4 (7.1) | 50.7 (7.1) | 52.1 (6.9) | 52.9 (9.6) | 54.3 (9.8) | 55.9 (9.6) |
| BMI, kg/m^2^ | 25.3 (5.0) | 25.2 (4.6) | 24.6 (4.2) | 25.9 (3.5) | 25.6 (3.3) | 25.2 (3.2) |
| Physical activity, MET-h/wk | 9.9 (12.7) | 12.4 (14.7) | 15.7 (17.5) | 16.0 (22.7) | 20.3 (29.3) | 26.6 (33.9) |
| Regular aspirin use^a^, % | 40.6 | 41.1 | 38.6 | 20.5 | 20.6 | 19.0 |
| Diet intake |  |  |  |  |  |  |
| Calorie intake, kcal/d | 1883 (501) | 1736 (524) | 1623 (509) | 2084 (615) | 1972 (628) | 1905 (600) |
| Alcohol, g/d | 7.6 (14.3) | 6.6 (10.5) | 6.6 (8.3) | 13.6 (20.1) | 10.7 (14.3) | 9.9 (10.6) |
| Processed meat, servings/wk | 3.1 (2.9) | 2.2 (2.1) | 1.3 (1.5) | 3.9 (3.7) | 2.6 (2.7) | 1.4 (1.7) |
| Red meat, servings/wk | 5.8 (2.9) | 4.5 (2.6) | 3.1 (2.1) | 5.9 (3.6) | 4.3 (2.9) | 2.7 (2.2) |
| Poultry, servings/wk | 1.6 (1.3) | 2.1 (1.6) | 2.7 (2.0) | 2.0 (1.6) | 2.5 (1.9) | 2.9 (2.2) |
| Fish, servings/wk | 1.4 (1.1) | 2.1 (1.7) | 3.0 (2.1) | 1.9 (1.7) | 2.9 (2.2) | 3.8 (2.6) |
| Whole grain, servings/wk | 5.0 (6.1) | 6.3 (6.8) | 8.1 (8.0) | 7.5 (7.9) | 9.8 (9.2) | 12.8 (11.1) |
| Refined carbohydrates, servings/wk | 10.9 (8.2) | 8.2 (6.6) | 6.1 (5.2) | 10.6 (8.8) | 8.5 (7.2) | 6.9 (5.9) |
| Fruits, servings/wk | 6.9 (5.0) | 9.5 (6.6) | 13.2 (8.7) | 7.4 (5.7) | 10.7 (8.0) | 15.7 (10.8) |
| Vegetables, servings/wk | 12.4 (6.8) | 16.2 (9.1) | 21.3 (12.6) | 16.4 (12.9) | 21.3 (14.3) | 27.4 (16.3) |
| Green leafy vegetables, servings/wk | 4.3 (3.2) | 5.7 (4.2) | 7.5 (5.5) | 3.8 (3.1) | 5.1 (4.0) | 6.7 (5.0) |
| Dark yellow vegetables, servings/wk | 1.6 (1.4) | 2.1 (2.0) | 2.8 (2.8) | 0.2 (0.4) | 0.3 (0.6) | 0.4 (0.8) |
| Cruciferous vegetables, servings/wk | 2.4 (1.8) | 3.1 (2.4) | 4.1 (3.5) | 1.7 (1.6) | 2.3 (2.2) | 3.2 (2.9) |
| Other vegetables, servings/wk | 4.1 (3.2) | 5.3 (4.1) | 6.8 (5.7) | 10.7 (11.4) | 13.5 (12.0) | 17.1 (12.9) |
| High-fat dairy, servings/wk | 8.8 (8.3) | 7.6 (7.1) | 6.4 (6.3) | 8.2 (8.4) | 6.9 (7.1) | 5.3 (5.6) |
| Low-fat dairy, servings/wk | 5.7 (6.8) | 6.4 (6.7) | 7.0 (6.6) | 6.7 (8.1) | 7.1 (7.4) | 7.2 (7.1) |
| Nuts, servings/wk | 1.4 (2.0) | 2.0 (2.9) | 3.0 (4.3) | 2.2 (3.1) | 3.3 (4.3) | 4.7 (6.0) |
| Coffee, servings/wk | 17.4 (13.9) | 17.7 (13.5) | 17.5 (13.5) | 30.5 (20.6) | 29.7 (20.4) | 27.7 (20.4) |
| SSB, servings/wk | 3.5 (5.5) | 1.8 (3.3) | 0.8 (2.2) | 3.9 (5.4) | 2.2 (3.5) | 1.2 (2.4) |
| Abbreviation: NHS, nurses' health study; HPFS, health professional follow-up study; BMI, body mass index; MET, metabolic equivalent task; SSB, sugar sweetened beverage; AHEI-2010, alternate healthy eating index-2010; aMED, alternate Mediterranean diet; DASH, dietary approaches to stop hypertension; EDIP, empirical dietary inflammatory pattern; EDIR, empirical dietary index for insulin resistance; EDIH, empirical dietary index for hyperinsulinemia; BMI, body mass index.  ^a^ Updated information over the follow-up due to no baseline information in men.  ^b^ Age was not standardized.  Data were presented as mean±SD (unless otherwise specified). | | | | | | |

| **(Continued) Table S1. Age-standardized baseline characteristics of participants according to tertile of dietary patterns** | | | | | | |
| --- | --- | --- | --- | --- | --- | --- |
|  | **NHS (69,751 women)** | | | **HPFS (47,232 Men)** | | |
| **aMED** | **Tertile 1** | **Tertile 2** | **Tertile 3** | **Tertile 1** | **Tertile 2** | **Tertile 3** |
| Age, year^b^ | 49.5 (7.2) | 50.5 (7.1) | 51.8 (7.0) | 53.0 (9.7) | 54.7 (9.7) | 55.6 (9.7) |
| BMI, kg/m^2^ | 25.2 (4.8) | 25.1 (4.7) | 24.9 (4.4) | 25.8 (3.4) | 25.6 (3.3) | 25.2 (3.2) |
| Physical activity, MET-h/wk | 9.8 (12.6) | 11.8 (14.4) | 15.3 (17.1) | 16.4 (24.8) | 20.7 (29.3) | 26.6 (33.2) |
| Regular aspirin use^a^, % | 39.3 | 40.7 | 40.3 | 20.0 | 20.1 | 19.8 |
| Diet intake |  |  |  |  |  |  |
| Calorie intake, kcal/d | 1548 (465) | 1693 (501) | 1935 (527) | 1802 (567) | 1994 (614) | 2208 (616) |
| Alcohol, g/d | 6.9 (13.0) | 6.9 (11.4) | 6.9 (9.8) | 12.0 (17.8) | 11.1 (15.3) | 11.0 (12.6) |
| Processed meat, servings/wk | 2.4 (2.6) | 2.2 (2.3) | 1.9 (2.1) | 3.2 (3.4) | 2.6 (3.0) | 1.9 (2.4) |
| Red meat, servings/wk | 4.8 (2.7) | 4.6 (2.8) | 4.2 (2.8) | 5.0 (3.3) | 4.4 (3.2) | 3.4 (2.8) |
| Poultry, servings/wk | 1.6 (1.2) | 2.0 (1.6) | 2.7 (1.9) | 2.0 (1.6) | 2.5 (2.0) | 3.0 (2.1) |
| Fish, servings/wk | 1.2 (1.0) | 1.9 (1.5) | 3.0 (2.1) | 1.8 (1.4) | 2.8 (2.1) | 4.1 (2.7) |
| Whole grain, servings/wk | 3.1 (4.1) | 5.5 (6.2) | 9.7 (8.1) | 5.9 (6.7) | 10.1 (9.3) | 14.8 (11.0) |
| Refined carbohydrates, servings/wk | 8.9 (7.4) | 8.6 (7.2) | 8.0 (6.7) | 9.1 (8.0) | 8.5 (7.6) | 8.3 (6.9) |
| Fruits, servings/wk | 5.8 (4.5) | 8.7 (6.4) | 13.6 (8.0) | 7.0 (6.0) | 11.2 (8.4) | 16.3 (10.3) |
| Vegetables, servings/wk | 10.0 (5.1) | 14.7 (8.2) | 22.8 (11.6) | 15.3 (12.9) | 21.7 (14.2) | 29.3 (15.5) |
| Green leafy vegetables, servings/wk | 3.7 (2.9) | 5.3 (4.0) | 7.8 (5.2) | 3.6 (3.1) | 5.2 (4.0) | 7.1 (4.9) |
| Dark yellow vegetables, servings/wk | 1.2 (1.1) | 1.8 (1.8) | 3.1 (2.6) | 0.2 (0.4) | 0.3 (0.6) | 0.5 (0.8) |
| Cruciferous vegetables, servings/wk | 2.0 (1.5) | 2.8 (2.3) | 4.4 (3.3) | 1.6 (1.4) | 2.4 (2.2) | 3.5 (3.0) |
| Other vegetables, servings/wk | 3.1 (2.3) | 4.7 (3.7) | 7.6 (5.5) | 10.0 (11.6) | 13.8 (12.0) | 18.2 (12.2) |
| High-fat dairy, servings/wk | 8.4 (8.5) | 7.5 (7.3) | 7.2 (6.5) | 7.8 (8.2) | 6.9 (7.1) | 5.6 (5.7) |
| Low-fat dairy, servings/wk | 4.9 (6.3) | 5.9 (6.5) | 7.8 (7.0) | 6.1 (7.5) | 7.1 (7.6) | 7.8 (7.3) |
| Nuts, servings/wk | 1.0 (1.8) | 2.0 (3.0) | 3.1 (3.9) | 2.0 (3.2) | 3.5 (4.7) | 5.0 (5.7) |
| Coffee, servings/wk | 17.8 (13.9) | 17.6 (13.7) | 17.2 (13.3) | 30.8 (20.4) | 29.2 (20.6) | 27.7 (20.5) |
| SSB, servings/wk | 2.6 (5.0) | 2.1 (4.2) | 1.7 (3.3) | 3.0 (4.9) | 2.4 (4.0) | 2.0 (3.3) |
| Abbreviation: NHS, nurses' health study; HPFS, health professional follow-up study; BMI, body mass index; MET, metabolic equivalent task; SSB, sugar sweetened beverage; AHEI-2010, alternate healthy eating index-2010; aMED, alternate Mediterranean diet; DASH, dietary approaches to stop hypertension; EDIP, empirical dietary inflammatory pattern; EDIR, empirical dietary index for insulin resistance; EDIH, empirical dietary index for hyperinsulinemia; BMI, body mass index; BMI, body mass index.  ^a^ Updated information over the follow-up due to no baseline information in men.  ^b^ Age was not standardized.  Data were presented as mean±SD (unless otherwise specified). | | | | | | |

| **(Continued) Table S1. Age-standardized baseline characteristics of participants according to tertile of dietary patterns** | | | | | | |
| --- | --- | --- | --- | --- | --- | --- |
|  | **NHS (69,751 women)** | | | **HPFS (47,232 Men)** | | |
| **DASH** | **Tertile 1** | **Tertile 2** | **Tertile 3** | **Tertile 1** | **Tertile 2** | **Tertile 3** |
| Age, year^b^ | 48.9 (7.0) | 50.8 (7.1) | 52.7 (6.9) | 52.4 (9.3) | 54.6 (9.7) | 56.1(9.9) |
| BMI, kg/m^2^ | 25.2 (5.0) | 25.1 (4.6) | 24.8 (4.3) | 25.9 (3.4) | 25.7 (3.3) | 25.1(3.3) |
| Physical activity, MET-h/wk | 9.6 (12.6) | 12.5 (14.7) | 16.2 (17.7) | 15.6 (23.4) | 20.3 (27.1) | 27.2(34.7) |
| Regular aspirin use^a^, % | 40.0 | 40.6 | 39.5 | 20.6 | 20.7 | 18.7 |
| Diet intake |  |  |  |  |  |  |
| Calorie intake, kcal/d | 1730 (529) | 1731 (534) | 1796 (505) | 1851 (591) | 1983 (624) | 2136 (613) |
| Alcohol, g/d | 7.6 (12.8) | 7.1 (11.2) | 5.9 (9.5) | 12.5 (16.8) | 11.5 (15.5) | 10.1 (13.9) |
| Processed meat, servings/wk | 3.0 (2.9) | 2.1 (2.1) | 1.3 (1.5) | 3.6 (3.5) | 2.7 (2.9) | 1.5 (2.0) |
| Red meat, servings/wk | 5.4 (2.9) | 4.5 (2.7) | 3.4 (2.3) | 5.3 (3.3) | 4.5 (3.2) | 3.2 (2.8) |
| Poultry, servings/wk | 1.7 (1.4) | 2.1 (1.6) | 2.7 (2.0) | 2.1 (1.7) | 2.5 (1.9) | 2.9 (2.2) |
| Fish, servings/wk | 1.6 (1.4) | 2.1 (1.6) | 2.9 (2.2) | 2.1 (1.7) | 2.8 (2.3) | 3.6 (2.7) |
| Whole grain, servings/wk | 3.4 (4.7) | 6.2 (6.4) | 10.3 (8.3) | 5.0 (5.7) | 9.5 (8.2) | 15.7 (11.3) |
| Refined carbohydrates, servings/wk | 10.8 (8.2) | 8.2 (6.6) | 6.3 (5.4) | 9.8 (8.2) | 8.6 (7.4) | 7.6 (6.7) |
| Fruits, servings/wk | 5.7 (4.5) | 9.5 (6.2) | 14.9 (8.2) | 6.3 (5.0) | 10.5 (6.8) | 17.1 (10.8) |
| Vegetables, servings/wk | 11.2 (6.4) | 16.2 (8.8) | 23.2 (12.3) | 15.9 (12.7) | 21.2 (14.0) | 28.1 (16.2) |
| Green leafy vegetables, servings/wk | 4.0 (3.2) | 5.8 (4.2) | 8.0 (5.5) | 3.6 (3.0) | 5.1 (3.8) | 6.9 (5.1) |
| Dark yellow vegetables, servings/wk | 1.3 (1.3) | 2.0 (1.8) | 3.2 (2.9) | 0.2 (0.4) | 0.3 (0.5) | 0.4 (0.8) |
| Cruciferous vegetables, servings/wk | 2.2 (1.7) | 3.1 (2.4) | 4.5 (3.6) | 1.6 (1.5) | 2.3 (2.0) | 3.4 (3.0) |
| Other vegetables, servings/wk | 3.7 (3.0) | 5.3 (4.1) | 7.5 (5.7) | 10.5 (11.4) | 13.5 (12.0) | 17.4 (12.7) |
| High-fat dairy, servings/wk | 8.6 (8.4) | 7.5(7.1) | 6.6 (6.2) | 7.6 (8.0) | 6.9 (7.0) | 5.9 (6.4) |
| Low-fat dairy, servings/wk | 3.1 (4.8) | 6.3 (6.3) | 10.1 (7.3) | 3.7 (5.3) | 6.9 (7.2) | 10.3 (8.3) |
| Nuts, servings/wk | 1.3 (2.5) | 2.1 (3.1) | 3.1 (4.0) | 2.1 (3.1) | 3.3 (4.5) | 4.8 (5.8) |
| Coffee, servings/wk | 18.2 (14.0) | 17.6 (13.5) | 16.6 (13.3) | 31.6 (19.9) | 29.8 (20.4) | 26.4 (20.9) |
| SSB, servings/wk | 3.6 (5.5) | 1.7 (3.2) | 0.8 (2.1) | 3.7 (5.1) | 2.3 (3.8) | 1.3 (2.9) |
| Abbreviation: NHS, nurses' health study; HPFS, health professional follow-up study; BMI, body mass index; MET, metabolic equivalent task; SSB, sugar sweetened beverage; AHEI-2010, alternate healthy eating index-2010; aMED, alternate Mediterranean diet; DASH, dietary approaches to stop hypertension; EDIP, empirical dietary inflammatory pattern; EDIR, empirical dietary index for insulin resistance; EDIH, empirical dietary index for hyperinsulinemia; BMI, body mass index.  ^a^ Updated information over the follow-up due to no baseline information in men.  ^b^ Age was not standardized.  Data were presented as mean±SD (unless otherwise specified). | | | | | | |

| **(Continued) Table S1. Age-standardized baseline characteristics of participants according to tertile of dietary patterns** | | | | | | |
| --- | --- | --- | --- | --- | --- | --- |
|  | **NHS (69,751 women)** | | | **HPFS (47,232 Men)** | | |
| **Prudent** | **Tertile 1** | **Tertile 2** | **Tertile 3** | **Tertile 1** | **Tertile 2** | **Tertile 3** |
| Age, year^b^ | 49.3 (7.0) | 50.7 (7.1) | 52.2 (7.0) | 53.0 (9.6) | 54.5 (9.7) | 55.6 (9.7) |
| BMI, kg/m^2^ | 24.9 (4.7) | 25.0 (4.6) | 25.2 (4.6) | 25.7 (3.3) | 25.5 (3.3) | 25.5 (3.4) |
| Physical activity, MET-h/wk | 10.2 (13.3) | 12.4 (14.7) | 15.4 (17.2) | 16.0 (23.6) | 20.7 (28.6) | 26.1 (33.9) |
| Regular aspirin use^a^, % | 39.1 | 40.9 | 40.3 | 20.2 | 20.5 | 19.3 |
| Diet intake |  |  |  |  |  |  |
| Calorie intake, kcal/d | 1525 (478) | 1734 (482) | 1990 (512) | 1704 (535) | 1965 (553) | 2297 (624) |
| Alcohol, g/d | 8.7 (13.6) | 6.6 (10.6) | 5.6 (9.0) | 11.7 (16.3) | 11.4 (15.4) | 11.0 (14.5) |
| Processed meat, servings/wk | 2.4 (2.6) | 2.2 (2.2) | 2.0 (2.1) | 3.0 (3.3) | 2.6 (2.9) | 2.2 (2.7) |
| Red meat, servings/wk | 4.4 (2.7) | 4.6 (2.7) | 4.5 (2.8) | 4.6 (3.3) | 4.4 (3.2) | 4.0 (3.2) |
| Poultry, servings/wk | 1.6 (1.3) | 2.1 (1.5) | 2.8 (2.0) | 1.7 (1.3) | 2.5 (1.6) | 3.3 (2.4) |
| Fish, servings/wk | 1.4 (1.2) | 2.0 (1.5) | 3.1 (2.2) | 1.7 (1.3) | 2.7 (1.8) | 4.1 (3.0) |
| Whole grain, servings/wk | 3.8 (5.2) | 6.1 (6.3) | 9.6 (8.3) | 6.1 (6.3) | 9.8 (8.5) | 14.2 (11.7) |
| Refined carbohydrates, servings/wk | 8.6 (7.5) | 8.5 (7.1) | 8.1 (6.7) | 8.5 (7.7) | 8.6 (7.4) | 8.9 (7.5) |
| Fruits, servings/wk | 5.6 (4.6) | 9.5 (5.8) | 14.6 (8.2) | 6.4 (4.7) | 10.5 (6.5) | 16.9 (11.2) |
| Vegetables, servings/wk | 9.6 (4.9) | 15.5 (6.4) | 24.9 (12.0) | 12.7 (11.3) | 20.0 (11.4) | 32.4 (15.5) |
| Green leafy vegetables, servings/wk | 3.7 (3.0) | 5.6 (3.7) | 8.3 (5.5) | 2.7 (2.0) | 4.8 (2.7) | 8.1 (5.4) |
| Dark yellow vegetables, servings/wk | 1.0 (1.1) | 2.0 (1.6) | 3.5 (2.8) | 0.2 (0.3) | 0.3 (0.4) | 0.5 (0.9) |
| Cruciferous vegetables, servings/wk | 1.8 (1.4) | 3.0 (2.0) | 4.8 (3.5) | 1.3 (1.0) | 2.1 (1.6) | 3.8 (3.1) |
| Other vegetables, servings/wk | 3.0 (2.3) | 5.0 (3.4) | 8.3 (5.8) | 8.5 (10.9) | 12.8 (10.7) | 20.0 (12.6) |
| High-fat dairy, servings/wk | 7.3 (7.7) | 7.6 (7.2) | 7.8 (7.1) | 6.9 (7.5) | 6.9 (7.0) | 6.6 (7.0) |
| Low-fat dairy, servings/wk | 4.0 (5.4) | 6.3 (6.3) | 8.9 (7.4) | 5.3 (6.5) | 7.0 (7.4) | 8.5 (8.2) |
| Nuts, servings/wk | 1.6 (2.6) | 2.1 (3.1) | 2.8 (3.9) | 2.5 (3.5) | 3.4 (4.6) | 4.3 (5.8) |
| Coffee, servings/wk | 18.4 (14.0) | 17.3 (13.4) | 16.7 (13.3) | 30.6 (20.3) | 29.2 (20.4) | 28.0 (20.8) |
| SSB, servings/wk | 2.6 (5.0) | 2.0 (3.8) | 1.6 (3.2) | 3.0 (4.9) | 2.3 (3.8) | 2.0 (3.6) |
| Abbreviation: NHS, nurses' health study; HPFS, health professional follow-up study; BMI, body mass index; MET, metabolic equivalent task; SSB, sugar sweetened beverage; AHEI-2010, alternate healthy eating index-2010; aMED, alternate Mediterranean diet; DASH, dietary approaches to stop hypertension; EDIP, empirical dietary inflammatory pattern; EDIR, empirical dietary index for insulin resistance; EDIH, empirical dietary index for hyperinsulinemia; BMI, body mass index.  ^a^ Updated information over the follow-up due to no baseline information in men.  ^b^ Age was not standardized.  Data were presented as mean±SD (unless otherwise specified). | | | | | | |

| **(Continued) Table S1. Age-standardized baseline characteristics of participants according to tertile of dietary patterns** | | | | | | |
| --- | --- | --- | --- | --- | --- | --- |
|  | **NHS (69,751 women)** | | | **HPFS (47,232 Men)** | | |
| **Western** | **Tertile 1** | **Tertile 2** | **Tertile 3** | **Tertile 1** | **Tertile 2** | **Tertile 3** |
| Age, year^b^ | 52.7 (6.8) | 50.7 (7.1) | 48.9 (7.1) | 55.9 (9.8) | 54.2 (9.7) | 53.1 (9.5) |
| BMI, kg/m^2^ | 24.6 (4.3) | 25.0 (4.6) | 25.4 (5.0) | 25.2 (3.2) | 25.6 (3.3) | 25.9 (3.4) |
| Physical activity, MET-h/wk | 14.7 (17.1) | 12.4 (14.8) | 11.1 (13.6) | 24.4 (34.2) | 20.2 (27.4) | 18.3 (26.0) |
| Regular aspirin use^a^, % | 36.9 | 40.8 | 42.8 | 17.6 | 20.6 | 22.1 |
| Diet intake |  |  |  |  |  |  |
| Calorie intake, kcal/d | 1370 (379) | 1697 (376) | 2177 (466) | 1561 (444) | 1903 (433) | 2498 (562) |
| Alcohol, g/d | 7.2 (11.6) | 7.1 (11.3) | 6.4 (10.7) | 8.1 (11.5) | 11.6 (15.1) | 14.5 (18.5) |
| Processed meat, servings/wk | 1.0 (1.3) | 2.1 (1.9) | 3.4 (2.9) | 1.0 (1.1) | 2.3 (1.8) | 4.6 (3.9) |
| Red meat, servings/wk | 3.0 (2.0) | 4.4 (2.3) | 6.1 (3.0) | 2.2 (1.6) | 4.2 (2.3) | 6.6 (3.6) |
| Poultry, servings/wk | 2.3 (1.9) | 2.0 (1.6) | 2.1 (1.5) | 2.6 (2.1) | 2.4 (1.8) | 2.4 (1.9) |
| Fish, servings/wk | 2.5 (2.2) | 2.0 (1.7) | 2.0 (1.5) | 3.3 (2.7) | 2.8 (2.1) | 2.5 (2.0) |
| Whole grain, servings/wk | 6.8 (7.6) | 6.3 (6.9) | 6.3 (6.9) | 11.0 (10.6) | 9.6 (9.1) | 9.4 (9.3) |
| Refined carbohydrates, servings/wk | 4.9 (4.5) | 8.1 (6.1) | 12.2 (8.1) | 5.5 (4.4) | 7.8 (5.9) | 12.7 (9.5) |
| Fruits, servings/wk | 10.6 (8.2) | 9.5 (7.1) | 9.6 (6.8) | 13.4 (10.7) | 10.6 (8.2) | 9.8 (7.8) |
| Vegetables, servings/wk | 17.5 (12.1) | 16.0 (9.7) | 16.5 (9.4) | 22.5 (16.2) | 21.4 (15.0) | 21.2 (14.3) |
| Green leafy vegetables, servings/wk | 6.4 (5.3) | 5.7 (4.4) | 5.5 (4.1) | 5.3 (4.6) | 5.2 (4.2) | 5.2 (4.0) |
| Dark yellow vegetables, servings/wk | 2.2 (2.6) | 2.1 (2.0) | 2.2 (2.0) | 0.4 (0.7) | 0.3 (0.6) | 0.3 (0.6) |
| Cruciferous vegetables, servings/wk | 3.4 (3.2) | 3.1 (2.7) | 3.2 (2.5) | 2.7 (2.8) | 2.3 (2.1) | 2.3 (2.1) |
| Other vegetables, servings/wk | 5.5 (5.3) | 5.2 (4.2) | 5.7 (4.4) | 14.2 (12.9) | 13.6 (12.3) | 13.4 (11.7) |
| High-fat dairy, servings/wk | 5.5 (6.0) | 7.5 (6.9) | 9.9 (8.2) | 3.7 (3.4) | 6.3 (5.4) | 10.5 (9.6) |
| Low-fat dairy, servings/wk | 7.0 (7.1) | 6.2 (6.6) | 6.0 (6.5) | 7.2 (7.5) | 6.9 (7.3) | 6.8 (7.8) |
| Nuts, servings/wk | 1.7 (2.8) | 2.1 (3.1) | 2.7 (3.8) | 2.5 (3.5) | 3.3 (4.3) | 4.5 (6.0) |
| Coffee, servings/wk | 16.8 (13.5) | 17.5 (13.5) | 18.3 (13.8) | 23.0 (19.9) | 29.8 (20.0) | 35.1 (19.8) |
| SSB, servings/wk | 0.9 (2.7) | 1.8 (3.6) | 3.4 (5.1) | 1.1 (2.1) | 2.2 (3.3) | 4.0 (5.6) |
| Abbreviation: NHS, nurses' health study; HPFS, health professional follow-up study; BMI, body mass index; MET, metabolic equivalent task; SSB, sugar sweetened beverage; AHEI-2010, alternate healthy eating index-2010; aMED, alternate Mediterranean diet; DASH, dietary approaches to stop hypertension; EDIP, empirical dietary inflammatory pattern; EDIR, empirical dietary index for insulin resistance; EDIH, empirical dietary index for hyperinsulinemia; BMI, body mass index.  ^a^ Updated information over the follow-up due to no baseline information in men.  ^b^ Age was not standardized.  Data were presented as mean±SD (unless otherwise specified). | | | | | | |

| **(Continued) Table S1. Age-standardized baseline characteristics of participants according to tertile of dietary patterns** | | | | | | |
| --- | --- | --- | --- | --- | --- | --- |
|  | **NHS (69,751 women)** | | | **HPFS (47,232 Men)** | | |
| **EDIP** | **Tertile 1** | **Tertile 2** | **Tertile 3** | **Tertile 1** | **Tertile 2** | **Tertile 3** |
| Age, year^b^ | 51.1 (6.9) | 51.0 (7.2) | 50.1 (7.2) | 53.7 (9.3) | 54.9 (9.9) | 54.5 (10.0) |
| BMI, kg/m^2^ | 24.0 (3.8) | 24.8 (4.3) | 26.2 (5.4) | 25.3 (3.1) | 25.4 (3.2) | 25.9 (3.6) |
| Physical activity, MET-h/wk | 13.8 (16.2) | 12.6 (14.9) | 11.7 (14.6) | 22.5 (30.1) | 20.4 (27.6) | 19.5 (29.5) |
| Regular aspirin use^a^, % | 39.3 | 39.6 | 41.5 | 22.1 | 19.3 | 18.8 |
| Diet intake |  |  |  |  |  |  |
| Calorie intake, kcal/d | 1694 (500) | 1678 (497) | 1874 (547) | 1953 (592) | 1873 (579) | 2143 (656) |
| Alcohol, g/d | 11.0 (14.0) | 5.5 (9.0) | 4.2 (8.6) | 17.8 (18.9) | 9.2 (12.3) | 7.0 (11.9) |
| Processed meat, servings/wk | 1.7 (1.7) | 2.0 (1.9) | 2.9 (3.0) | 2.0 (2.1) | 2.2 (2.4) | 3.5 (3.9) |
| Red meat, servings/wk | 3.9 (2.4) | 4.2 (2.5) | 5.3 (3.1) | 3.7 (2.7) | 4.0 (2.9) | 5.3 (3.8) |
| Poultry, servings/wk | 2.1 (1.7) | 2.1 (1.6) | 2.2 (1.8) | 2.4 (1.9) | 2.4 (1.8) | 2.6 (2.1) |
| Fish, servings/wk | 2.0 (1.6) | 2.1 (1.7) | 2.4 (2.1) | 2.6 (2.0) | 2.7 (2.1) | 3.2 (2.8) |
| Whole grain, servings/wk | 7.2 (7.6) | 6.5 (7.0) | 5.7 (6.7) | 10.8 (10.2) | 10.1 (9.6) | 9.2 (9.4) |
| Refined carbohydrates, servings/wk | 6.4 (5.0) | 7.7 (6.1) | 11.2 (8.6) | 6.6 (5.3) | 7.6 (6.2) | 11.6 (9.4) |
| Fruits, servings/wk | 10.0 (7.6) | 9.9 (7.2) | 9.8 (7.6) | 11.2 (9.5) | 11.3 (8.8) | 11.4 (9.2) |
| Vegetables, servings/wk | 18.1 (11.4) | 15.9 (9.3) | 16.0 (10.4) | 22.6 (15.6) | 20.8 (14.5) | 21.6 (15.5) |
| Green leafy vegetables, servings/wk | 7.2 (5.8) | 5.5 (3.8) | 4.9 (3.6) | 6.2 (5.2) | 4.9 (3.7) | 4.5 (3.6) |
| Dark yellow vegetables, servings/wk | 2.5 (2.7) | 2.1 (1.9) | 1.9 (1.7) | 0.3 (0.8) | 0.3 (0.6) | 0.3 (0.6) |
| Cruciferous vegetables, servings/wk | 3.3 (3.0) | 3.1 (2.6) | 3.2 (2.8) | 2.5 (2.5) | 2.4 (2.2) | 2.4 (2.5) |
| Other vegetables, servings/wk | 5.2 (4.1) | 5.2 (4.0) | 6.0 (5.6) | 13.6 (12.2) | 13.2 (12.0) | 14.4 (12.8) |
| High-fat dairy, servings/wk | 7.7 (7.8) | 7.4 (7.1) | 7.6 (7.0) | 6.7 (7.3) | 6.4 (6.9) | 7.2 (7.3) |
| Low-fat dairy, servings/wk | 6.4 (6.6) | 6.6 (6.8) | 6.3 (6.9) | 6.8 (7.3) | 7.0 (7.3) | 7.1 (8.0) |
| Nuts, servings/wk | 2.3 (3.6) | 2.1 (3.1) | 2.1 (3.1) | 3.6 (5.1) | 3.3 (4.4) | 3.4 (4.7) |
| Coffee, servings/wk | 25.9 (13.8) | 16.1 (11.5) | 10.4 (10.3) | 38.7 (18.6) | 27.6 (19.4) | 21.3 (19.7) |
| SSB, servings/wk | 1.0 (1.9) | 1.6 (2.5) | 3.7 (6.0) | 1.3 (2.3) | 1.9 (2.9) | 4.2 (6.0) |
| Abbreviation: NHS, nurses' health study; HPFS, health professional follow-up study; BMI, body mass index; MET, metabolic equivalent task; SSB, sugar sweetened beverage; AHEI-2010, alternate healthy eating index-2010; aMED, alternate Mediterranean diet; DASH, dietary approaches to stop hypertension; EDIP, empirical dietary inflammatory pattern; EDIR, empirical dietary index for insulin resistance; EDIH, empirical dietary index for hyperinsulinemia; BMI, body mass index.  ^a^ Updated information over the follow-up due to no baseline information in men.  ^b^ Age was not standardized.  Data were presented as mean±SD (unless otherwise specified). | | | | | | |

| **(Continued) Table S1. Age-standardized baseline characteristics of participants according to tertile of dietary patterns** | | | | | | |
| --- | --- | --- | --- | --- | --- | --- |
|  | **NHS (69,751 women)** | | | **HPFS (47,232 Men)** | | |
| **EDIR** | **Tertile 1** | **Tertile 2** | **Tertile 3** | **Tertile 1** | **Tertile 2** | **Tertile 3** |
| Age, year^b^ | 51.6 (6.9) | 50.8 (7.2) | 49.8 (7.2) | 55.0 (9.6) | 54.3 (9.8) | 53.8 (9.9) |
| BMI, kg/m^2^ | 23.9 (3.7) | 24.9 (4.4) | 26.3 (5.4) | 25.3 (3.1) | 25.5 (3.2) | 25.9 (3.6) |
| Physical activity, MET-h/wk | 13.7 (16.3) | 12.7 (15.0) | 11.6 (14.2) | 21.2 (29.7) | 20.7 (28.2) | 20.6 (29.2) |
| Regular aspirin use^a^, % | 38.4 | 39.8 | 42.3 | 19.8 | 19.9 | 20.4 |
| Diet intake |  |  |  |  |  |  |
| Calorie intake, kcal/d | 1564 (479) | 1673 (465) | 2010 (516) | 1838 (572) | 1889 (561) | 2237 (642) |
| Alcohol, g/d | 12.8 (15.5) | 4.6 (7.1) | 3.2 (5.6) | 20.5 (20.2) | 8.0 (10.2) | 5.4 (8.3) |
| Processed meat, servings/wk | 1.4 (1.4) | 1.9 (1.7) | 3.3 (3.1) | 1.9 (2.0) | 2.3 (2.3) | 3.6 (4.0) |
| Red meat, servings/wk | 3.3 (2.0) | 4.2 (2.3) | 6.0 (3.1) | 3.4 (2.4) | 4.0 (2.8) | 5.6 (3.9) |
| Poultry, servings/wk | 2.1 (1.6) | 2.1 (1.6) | 2.3 (1.8) | 2.3 (1.8) | 2.5 (1.9) | 2.7 (2.2) |
| Fish, servings/wk | 2.0 (1.6) | 2.1 (1.8) | 2.4 (2.0) | 2.6 (2.1) | 2.8 (2.1) | 3.1 (2.7) |
| Whole grain, servings/wk | 6.3 (7.1) | 6.5 (7.1) | 6.7 (7.2) | 9.3 (9.2) | 10.2 (9.8) | 10.6 (10.2) |
| Refined carbohydrates, servings/wk | 5.7 (4.5) | 7.4 (5.6) | 12.1 (8.6) | 6.3 (5.1) | 7.9 (6.3) | 11.7 (9.4) |
| Fruits, servings/wk | 9.1 (7.3) | 9.9 (7.3) | 10.6 (7.8) | 9.7 (8.1) | 11.3 (8.6) | 12.8 (10.2) |
| Vegetables, servings/wk | 15.8 (9.9) | 16.2 (9.9) | 18.0 (11.5) | 16.7 (11.0) | 20.0 (12.4) | 28.4 (18.7) |
| Green leafy vegetables, servings/wk | 6.0 (5.0) | 5.7 (4.3) | 5.9 (4.5) | 4.8 (4.2) | 5.0 (3.9) | 5.7 (4.6) |
| Dark yellow vegetables, servings/wk | 2.1 (2.5) | 2.1 (2.0) | 2.2 (2.0) | 0.3 (0.6) | 0.3 (0.6) | 0.4 (0.7) |
| Cruciferous vegetables, servings/wk | 3.0 (2.7) | 3.2 (2.7) | 3.5 (3.0) | 2.2 (2.1) | 2.4 (2.3) | 2.7 (2.6) |
| Other vegetables, servings/wk | 4.7 (3.6) | 5.2 (4.1) | 6.4 (5.8) | 9.4 (8.0) | 12.3 (9.3) | 19.6 (15.9) |
| High-fat dairy, servings/wk | 8.5 (9.1) | 7.0 (6.5) | 7.2 (5.9) | 7.5 (8.6) | 6.3 (6.4) | 6.7 (6.3) |
| Low-fat dairy, servings/wk | 5.7 (6.3) | 6.5 (6.8) | 7.0 (7.2) | 6.1 (7.0) | 6.9 (7.3) | 7.9 (8.1) |
| Nuts, servings/wk | 2.4 (4.0) | 2.0 (2.9) | 2.1 (2.8) | 3.8 (5.8) | 3.1 (4.1) | 3.3 (4.1) |
| Coffee, servings/wk | 23.5 (13.9) | 16.1 (12.6) | 13.0 (12.0) | 40.1 (17.1) | 27.5 (19.4) | 20.3 (19.8) |
| SSB, servings/wk | 1.5 (3.5) | 2.0 (3.9) | 2.7 (4.7) | 1.9 (3.6) | 2.4 (3.9) | 3.1 (4.9) |
| Abbreviation: NHS, nurses' health study; HPFS, health professional follow-up study; BMI, body mass index; MET, metabolic equivalent task; SSB, sugar sweetened beverage; AHEI-2010, alternate healthy eating index-2010; aMED, alternate Mediterranean diet; DASH, dietary approaches to stop hypertension; EDIP, empirical dietary inflammatory pattern; EDIR, empirical dietary index for insulin resistance; EDIH, empirical dietary index for hyperinsulinemia; BMI, body mass index.  ^a^ Updated information over the follow-up due to no baseline information in men.  ^b^ Age was not standardized.  Data were presented as mean±SD (unless otherwise specified). | | | | | | |

| **(Continued) Table S1. Age-standardized baseline characteristics of participants according to tertile of dietary patterns** | | | | | | |
| --- | --- | --- | --- | --- | --- | --- |
|  | **NHS (69,751 women)** | | | **HPFS (47,232 Men)** | | |
| **EDIH** | **Tertile 1** | **Tertile 2** | **Tertile 3** | **Tertile 1** | **Tertile 2** | **Tertile 3** |
| Age, year^b^ | 52.0 (6.9) | 50.9 (7.1) | 49.4 (7.1) | 55.9 (9.7) | 54.6 (9.7) | 52.7 (9.5) |
| BMI, kg/m^2^ | 24.0 (3.8) | 25.0 (4.4) | 26.1 (5.3) | 25.1 (3.1) | 25.5 (3.2) | 26.0 (3.6) |
| Physical activity, MET-h/wk | 14.1 (16.5) | 12.7 (15.0) | 11.3 (14.0) | 23.5 (31.4) | 20.7 (29.6) | 18.6 (25.9) |
| Regular aspirin use^a^, % | 37.4 | 40.2 | 42.8 | 18.7 | 19.7 | 21.7 |
| Diet intake |  |  |  |  |  |  |
| Calorie intake, kcal/d | 1490 (449) | 1685 (438) | 2073 (502) | 1712 (519) | 1889 (523) | 2366 (613) |
| Alcohol, g/d | 9.1 (12.7) | 6.0 (10.0) | 5.7 (10.6) | 13.2 (16.3) | 10.5 (14.4) | 10.6 (15.6) |
| Processed meat, servings/wk | 1.2 (1.2) | 1.9 (1.7) | 3.4 (3.1) | 1.3 (1.5) | 2.2 (2.0) | 4.3 (4.0) |
| Red meat, servings/wk | 3.0 (1.8) | 4.2 (2.2) | 6.3 (3.1) | 2.5 (1.9) | 3.9 (2.4) | 6.5 (3.6) |
| Poultry, servings/wk | 1.9 (1.4) | 2.1 (1.5) | 2.5 (2.0) | 2.2 (1.5) | 2.4 (1.7) | 2.8 (2.5) |
| Fish, servings/wk | 1.9 (1.6) | 2.2 (1.8) | 2.4 (2.1) | 2.7 (2.2) | 2.8 (2.2) | 3.0 (2.7) |
| Whole grain, servings/wk | 6.4 (7.1) | 6.5 (7.0) | 6.7 (7.3) | 10.4 (10.2) | 9.9 (9.5) | 9.8 (9.6) |
| Refined carbohydrates, servings/wk | 6.2 (5.4) | 8.1 (6.4) | 10.9 (8.2) | 7.0 (6.2) | 8.1 (6.9) | 10.8 (8.7) |
| Fruits, servings/wk | 10.5 (8.3) | 9.7 (7.0) | 9.4 (6.8) | 13.0 (11.0) | 10.7 (8.0) | 10.1 (7.7) |
| Vegetables, servings/wk | 17.0 (11.7) | 16.2 (9.7) | 16.8 (9.9) | 23.3 (16.2) | 20.8 (14.6) | 21.1 (14.7) |
| Green leafy vegetables, servings/wk | 6.4 (5.5) | 5.6 (4.1) | 5.6 (4.1) | 5.7 (4.9) | 5.0 (4.0) | 5.0 (3.9) |
| Dark yellow vegetables, servings/wk | 2.1 (2.4) | 2.1 (2.1) | 2.2 (2.1) | 0.3 (0.7) | 0.3 (0.6) | 0.3 (0.7) |
| Cruciferous vegetables, servings/wk | 3.1 (2.9) | 3.2 (2.7) | 3.4 (2.8) | 2.6 (2.6) | 2.3 (2.2) | 2.3 (2.2) |
| Other vegetables, servings/wk | 5.3 (5.0) | 5.3 (4.2) | 5.7 (4.5) | 14.7 (13.0) | 13.2 (11.9) | 13.4 (12.0) |
| High-fat dairy, servings/wk | 8.1 (8.9) | 7.0 (6.5) | 7.6 (6.3) | 6.6 (8.0) | 6.4 (6.5) | 7.5 (6.9) |
| Low-fat dairy, servings/wk | 5.6 (5.9) | 6.6 (6.8) | 7.0 (7.4) | 6.1 (6.6) | 7.1 (7.4) | 7.8 (8.5) |
| Nuts, servings/wk | 2.1 (3.5) | 2.1 (3.0) | 2.3 (3.3) | 3.2 (4.7) | 3.3 (4.5) | 3.8 (5.1) |
| Coffee, servings/wk | 22.0 (14.1) | 16.5 (12.9) | 14.2 (12.6) | 36.3 (19.2) | 27.4 (19.8) | 24.4 (20.4) |
| SSB, servings/wk | 0.9 (1.8) | 1.6 (2.7) | 3.7 (5.9) | 1.1 (1.9) | 2.0 (3.0) | 4.1 (5.8) |
| Abbreviation: NHS, nurses' health study; HPFS, health professional follow-up study; BMI, body mass index; MET, metabolic equivalent task; SSB, sugar sweetened beverage; AHEI-2010, alternate healthy eating index-2010; aMED, alternate Mediterranean diet; DASH, dietary approaches to stop hypertension; EDIP, empirical dietary inflammatory pattern; EDIR, empirical dietary index for insulin resistance; EDIH, empirical dietary index for hyperinsulinemia; BMI, body mass index.  ^a^ Updated information over the follow-up due to no baseline information in men.  ^b^ Age was not standardized.  Data were presented as mean±SD (unless otherwise specified). | | | | | | |

| **Table S2. Spearman correlations among dietary patterns^a^** | | | | | | | |  | | |
| --- | --- | --- | --- | --- | --- | --- | --- | --- | --- | --- |
|  | **AHEI-2010** | **aMED** | **DASH** | **Prudent** | **Western** | **EDIP** | **EDIR** | | **EDIH** |  |
| **Women** |  |  |  |  |  |  |  | |  |  |
| AHEI-2010 | 1.00 | 0.61 | 0.68 | 0.51 | -0.55 | -0.22 | -0.30 | | -0.42 |  |
| aMED |  | 1.00 | 0.73 | 0.80 | -0.12 | -0.09 | 0.05 | | -0.05 |  |
| DASH |  |  | 1.00 | 0.75 | -0.44 | -0.21 | -0.12 | | -0.30 |  |
| Prudent |  |  |  | 1.00 | -0.08 | -0.03 | 0.18 | | 0.02 |  |
| Western |  |  |  |  | 1.00 | 0.24 | 0.47 | | 0.63 |  |
| EDIP |  |  |  |  |  | 1.00 | 0.72 | | 0.64 |  |
| EDIR |  |  |  |  |  |  | 1.00 | | 0.76 |  |
| EDIH |  |  |  |  |  |  |  | | 1.00 |  |
| **Men** |  |  |  |  |  |  |  | |  |  |
| AHEI-2010 | 1.00 | 0.66 | 0.66 | 0.54 | -0.54 | -0.21 | -0.15 | | -0.45 |  |
| aMED |  | 1.00 | 0.75 | 0.78 | -0.21 | -0.11 | 0.07 | | -0.14 |  |
| DASH |  |  | 1.00 | 0.74 | -0.38 | -0.18 | 0.01 | | -0.28 |  |
| Prudent |  |  |  | 1.00 | -0.10 | -0.04 | 0.24 | | -0.02 |  |
| Western |  |  |  |  | 1.00 | 0.13 | 0.24 | | 0.63 |  |
| EDIP |  |  |  |  |  | 1.00 | 0.64 | | 0.57 |  |
| EDIR |  |  |  |  |  |  | 1.00 | | 0.64 |  |
| EDIH |  |  |  |  |  |  |  | | 1.00 |  |
| Abbreviation: AHEI-2010, alternate healthy eating index-2010; aMED, alternate Mediterranean diet; DASH, dietary approaches to stop hypertension; EDIP, empirical dietary inflammatory pattern; EDIR, empirical dietary index for insulin resistance; EDIH, empirical dietary index for hyperinsulinemia.  ^a^ Adjusted for age and body mass index. | | | | | | | | | |  |

| **Table S3. Summary of food items associated with the empirically derived dietary patterns**^a^ | | | |
| --- | --- | --- | --- |
|  | **EDIP** | **EDIR** | **EDIH** |
|  | **Inflammation** | **Insulin resistance** | **Hyperinsulinemia** |
| Red meat | **+** | **+** | **+** |
| Processed meat | **+** | **+** | **+** |
| Organ meat | **+** |  |  |
| Poultry |  |  | **+** |
| Non-fatty fish | **+** | **+** | **+** |
| Other vegetable | **+** | **+** |  |
| Refined grains | **+** | **+** |  |
| Margarine |  | **+** | **+** |
| Butter |  |  | **+** |
| Cream soup |  | **+** | **+** |
| High-fat dairy products |  | **-** | **-** |
| Low-fat dairy products |  |  | **+** |
| Eggs |  |  | **+** |
| High-energy beverages | **+** |  | **+** |
| Low-energy beverages | **+** | **+** | **+** |
| Tomatoes | **+** | **+** | **+** |
| Bear | **-** | **-** |  |
| Wine | **-** | **-** | **-** |
| Liquor |  | **-** |  |
| Tea | **-** |  |  |
| Coffee | **-** | **-** | **-** |
| Dark yellow vegetables | **-** | **-** |  |
| Leafy green vegetables | **-** | **-** | **-** |
| Whole fruits |  |  | **-** |
| Snacks | **-** |  |  |
| Fruit juice | **-** | **+** |  |
| Pizza | **-** |  |  |
| French fries |  |  | **+** |
| Nuts |  | **-** |  |
| Abbreviation: EDIP, empirical dietary inflammatory pattern; EDIR, empirical dietary index for insulin resistance; EDIH, empirical dietary index for hyperinsulinemia.  ^a^ 18 food items were associated with empirical models to predict inflammatory markers (EDIP), TG/HDL ratio (EDIR), and C-peptide (EDIH). ‘+’ indicates positive association and ‘-’ indicates an inverse association with indices. | | | |

| **Table S4. Association between cumulative average dietary pattern and multiple myeloma risk stratified by BMI** | | | | |
| --- | --- | --- | --- | --- |
|  | **Women** | | **Men** | |
|  | **HR (95% CI)**  **per 1-SD increase^a^** | **P for interaction^b^** | **HR (95% CI)**  **per 1-SD increase^a^** | **P for interaction^b^** |
| **AHEI-2010** |  |  |  |  |
| BMI<25 | 1.02 (0.83-1.26) | 0.82 | 0.96 (0.78-1.17) | 0.12 |
| BMI≥25 | 1.05 (0.86-1.28) |  | 1.12 (0.95-1.33) |  |
| **aMED** |  |  |  |  |
| BMI<25 | 0.96 (0.76-1.21) | 0.44 | 0.86 (0.70-1.07) | 0.10 |
| BMI≥25 | 1.00 (0.80-1.24) |  | 1.05 (0.88-1.25) |  |
| **DASH** |  |  |  |  |
| BMI<25 | 0.98 (0.78-1.22) | 0.90 | 0.85 (0.69-1.05) | 0.16 |
| BMI≥25 | 1.00 (0.81-1.22) |  | 1.01 (0.85-1.20) |  |
| **Prudent** |  |  |  |  |
| BMI<25 | 0.96 (0.75-1.23) | 0.68 | 0.89 (0.71-1.12) | 0.31 |
| BMI≥25 | 0.92 (0.74-1.15) |  | 1.02 (0.85-1.23) |  |
| **Western** |  |  |  |  |
| BMI<25 | 1.20 (0.88-1.64) | 0.74 | 1.18 (0.91-1.53) | 0.17 |
| BMI≥25 | 0.93 (0.71-1.23) |  | 0.91 (0.73-1.14) |  |
| **EDIP** |  |  |  |  |
| BMI<25 | 1.35 (1.06-1.73) | 0.007 | 1.25 (1.00-1.56) | 0.22 |
| BMI≥25 | 0.85 (0.69-1.03) |  | 1.09 (0.93-1.28) |  |
| **EDIR** |  |  |  |  |
| BMI<25 | 1.20 (0.93-1.56) | 0.14 | 1.19 (0.96-1.47) | 0.47 |
| BMI≥25 | 0.81 (0.65-1.00) |  | 1.06 (0.90-1.24) |  |
| **EDIH** |  |  |  |  |
| BMI<25 | 1.34 (1.02-1.76) | 0.30 | 1.23 (1.00-1.52) | 0.16 |
| BMI≥25 | 0.86 (0.68-1.09) |  | 1.03 (0.85-1.24) |  |
| Abbreviation: HR, hazard ratio; CI, confidence interval; SD, standard deviation; AHEI-2010, alternate healthy eating index-2010; aMED, alternate Mediterranean diet; DASH, dietary approaches to stop hypertension; EDIP, empirical dietary inflammatory pattern; EDIR, empirical dietary index for insulin resistance; EDIH, empirical dietary index for hyperinsulinemia; BMI, body mass index.  All models adjusted for age in years, cumulative average energy intake (continuous), and cumulative average BMI (continuous) (BMI was further adjusted within the strata).  ^a^ Sex-specific standard deviation was used.  ^b^ P for interaction between each dietary pattern (continuous) and BMI (binary). | | | | |

| **Table S5. Association between baseline dietary pattern and multiple myeloma risk in women and men** | | |
| --- | --- | --- |
|  | **HR (95% CI) per 1-SD increase^c^** | |
|  | **Women** | **Men** |
| **AHEI-2010** |  |  |
| Age- and energy-adjusted^a^ | 1.00 (0.86-1.15) | 1.07 (0.95-1.21) |
| Multivariable-adjusted^b^ | 1.01 (0.87-1.16) | 1.09 (0.96-1.23) |
| **aMED** |  |  |
| Age- and energy-adjusted^a^ | 0.95 (0.81-1.11) | 0.98 (0.86-1.11) |
| Multivariable-adjusted^b^ | 0.96 (0.82-1.12) | 0.99 (0.87-1.13) |
| **DASH** |  |  |
| Age- and energy-adjusted^a^ | 0.98 (0.85-1.14) | 0.96 (0.84-1.08) |
| Multivariable-adjusted^b^ | 0.99 (0.85-1.15) | 0.97 (0.85-1.10) |
| **Prudent** |  |  |
| Age- and energy-adjusted^a^ | 1.04 (0.88-1.24) | 0.98 (0.85-1.11) |
| Multivariable-adjusted^b^ | 1.04 (0.87-1.23) | 0.98 (0.86-1.12) |
| **Western** |  |  |
| Age- and energy-adjusted^a^ | 1.15 (0.94-1.41) | 1.05 (0.90-1.23) |
| Multivariable-adjusted^b^ | 1.13 (0.92-1.39) | 1.03 (0.88-1.20) |
| **EDIP** |  |  |
| Age- and energy-adjusted^a^ | 1.08 (0.92-1.28) | 1.21 (1.07-1.37) |
| Multivariable-adjusted^b^ | 1.03 (0.87-1.23) | 1.20 (1.06-1.36) |
| **EDIR** |  |  |
| Age- and energy-adjusted^a^ | 1.05 (0.89-1.25) | 1.16 (1.02-1.31) |
| Multivariable-adjusted^b^ | 1.00 (0.83-1.19) | 1.15 (1.01-1.30) |
| **EDIH** |  |  |
| Age- and energy-adjusted^a^ | 1.10 (0.91-1.33) | 1.22 (1.07-1.39) |
| Multivariable-adjusted^b^ | 1.04 (0.86-1.27) | 1.19 (1.05-1.36) |
| Abbreviation: HR, hazard ratio; CI, confidence interval; SD, standard deviation; AHEI-2010, alternate healthy eating index-2010; aMED, alternate Mediterranean diet; DASH, dietary approaches to stop hypertension; EDIP, empirical dietary inflammatory pattern; EDIR, empirical dietary index for insulin resistance; EDIH, empirical dietary index for hyperinsulinemia; BMI, body mass index.  ^a^ Adjusted for age in years and cumulative average energy intake (continuous).  ^b^ Additionally adjusted for cumulative average BMI (continuous).  ^c^ Sex-specific standard deviation was used. | | |

| **Table S6. Association between recent dietary pattern and multiple myeloma risk in women and men** | | |
| --- | --- | --- |
|  | **HR (95% CI) per 1-SD increase^c^** | |
|  | **Women** | **Men** |
| **AHEI-2010** |  |  |
| Age- and energy-adjusted^a^ | 0.94 (0.81-1.09) | 0.99 (0.86-1.15) |
| Multivariable-adjusted^b^ | 0.96 (0.83-1.11) | 1.01 (0.88-1.17) |
| **aMED** |  |  |
| Age- and energy-adjusted^a^ | 1.00 (0.86-1.16) | 1.00 (0.87-1.16) |
| Multivariable-adjusted^b^ | 1.02 (0.88-1.19) | 1.02 (0.88-1.19) |
| **DASH** |  |  |
| Age- and energy-adjusted^a^ | 0.97 (0.83-1.12) | 0.93 (0.80-1.07) |
| Multivariable-adjusted^b^ | 0.99 (0.85-1.15) | 0.95 (0.81-1.10) |
| **Prudent** |  |  |
| Age- and energy-adjusted^a^ | 0.87 (0.73-1.03) | 1.00 (0.86-1.17) |
| Multivariable-adjusted^b^ | 0.88 (0.74-1.04) | 1.02 (0.87-1.18) |
| **Western** |  |  |
| Age- and energy-adjusted^a^ | 1.09 (0.91-1.29) | 1.05 (0.88-1.24) |
| Multivariable-adjusted^b^ | 1.05 (0.88-1.25) | 1.02 (0.86-1.21) |
| **EDIP** |  |  |
| Age- and energy-adjusted^a^ | 0.99 (0.85-1.17) | 1.00 (0.86-1.15) |
| Multivariable-adjusted^b^ | 0.95 (0.80-1.12) | 0.98 (0.85-1.13) |
| **EDIR** |  |  |
| Age- and energy-adjusted^a^ | 0.98 (0.83-1.16) | 1.00 (0.88-1.13) |
| Multivariable-adjusted^b^ | 0.92 (0.77-1.10) | 0.98 (0.86-1.12) |
| **EDIH** |  |  |
| Age- and energy-adjusted^a^ | 1.13 (0.94-1.35) | 1.05 (0.90-1.22) |
| Multivariable-adjusted^b^ | 1.07 (0.89-1.29) | 1.02 (0.88-1.19) |
| Abbreviation: HR, hazard ratio; CI, confidence interval; SD, standard deviation; AHEI-2010, alternate healthy eating index-2010; aMED, alternate Mediterranean diet; DASH, dietary approaches to stop hypertension; EDIP, empirical dietary inflammatory pattern; EDIR, empirical dietary index for insulin resistance; EDIH, empirical dietary index for hyperinsulinemia; BMI, body mass index.  ^a^ Adjusted for age in years and cumulative average energy intake (continuous).  ^b^ Additionally adjusted for cumulative average BMI (continuous).  ^c^ Sex-specific standard deviation was used. | | |

| **Table S7. Association between tertile of cumulative average dietary pattern and multiple myeloma risk in women** | | | |
| --- | --- | --- | --- |
|  | **HR (95% CI) per 1-SD increase^c,d^** | | |
|  | **Tertile 1** | **Tertile 2** | **Tertile 3** |
| ***Presumed healthy dietary patterns***^e^ |  |  |  |
| **AHEI-2010** |  |  |  |
| Age- and energy-adjusted^a^ | 1 (reference) | 1.11 (0.80-1.56) | 1.00 (0.71-1.41) |
| Multivariable-adjusted^b^ | 1 (reference) | 1.12 (0.80-1.57) | 1.03 (0.73-1.46) |
| **aMED** |  |  |  |
| Age- and energy-adjusted^a^ | 1 (reference) | 1.03 (0.74-1.45) | 1.03 (0.72-1.48) |
| Multivariable-adjusted^b^ | 1 (reference) | 1.05 (0.75-1.47) | 1.07 (0.75-1.54) |
| **DASH** |  |  |  |
| Age- and energy-adjusted^a^ | 1 (reference) | 0.92 (0.65-1.28) | 0.96 (0.68-1.34) |
| Multivariable-adjusted^b^ | 1 (reference) | 0.92 (0.66-1.30) | 0.98 (0.70-1.38) |
| **Prudent** |  |  |  |
| Age- and energy-adjusted^a^ | 1 (reference) | 0.87 (0.62-1.22) | 0.90 (0.62-1.29) |
| Multivariable-adjusted^b^ | 1 (reference) | 0.87 (0.62-1.22) | 0.90 (0.62-1.29) |
| ***Presumed unhealthy dietary patterns*^e^** |  |  |  |
| **Western** |  |  |  |
| Age- and energy-adjusted^a^ | 1 (reference) | 1.37 (0.98-1.93) | 1.13 (0.73-1.75) |
| Multivariable-adjusted^b^ | 1 (reference) | 1.34 (0.96-1.88) | 1.07 (0.69-1.66) |
| **EDIP** |  |  |  |
| Age- and energy-adjusted^a^ | 1 (reference) | 1.10 (0.79-1.54) | 1.26 (0.90-1.77) |
| Multivariable-adjusted^b^ | 1 (reference) | 1.06 (0.76-1.49) | 1.16 (0.82-1.64) |
| **EDIR** |  |  |  |
| Age- and energy-adjusted^a^ | 1 (reference) | 1.12 (0.81-1.54) | 1.01 (0.70-1.44) |
| Multivariable-adjusted^b^ | 1 (reference) | 1.06 (0.77-1.47) | 0.89 (0.61-1.30) |
| **EDIH** |  |  |  |
| Age- and energy-adjusted^a^ | 1 (reference) | 1.16 (0.84-1.61) | 1.16 (0.80-1.69) |
| Multivariable-adjusted^b^ | 1 (reference) | 1.10 (0.79-1.53) | 1.04 (0.70-1.54) |
| Abbreviation: HR, hazard ratio; CI, confidence interval; SD, standard deviation; AHEI, alternate healthy eating index-2010; aMED, alternate Mediterranean diet; DASH, dietary approaches to stop hypertension; EDIP, empirical dietary inflammatory pattern; EDIR, empirical dietary index for insulin resistance; EDIH, empirical dietary index for hyperinsulinemia; BMI, body mass index.  ^a^ Adjusted for age in years and cumulative average energy intake (continuous).  ^b^ Additionally adjusted for cumulative average BMI (continuous).  ^c^ Sex-specific standard deviation was used.  ^d^ Cases/person-years: 215/1,709,737 for women and 263/1,082,520 for men.  ^e^ For the presumed healthy dietary patterns in the top half of the table, the reference group includes participants with a dietary pattern score in the lowest tertile (e.g., least adherence to the healthier dietary pattern). For the presumed unhealthy dietary patterns in the lower half of the table, the reference group includes participants with a dietary pattern score in the lowest tertile (e.g. least adherence to the less healthy dietary pattern). | | | |

| **Table S8. Association between tertile of cumulative average dietary pattern and multiple myeloma risk in men** | | | |
| --- | --- | --- | --- |
|  | **HR (95% CI) per 1-SD increase^c,d^** | | |
|  | **Tertile 1** | **Tertile 2** | **Tertile 3** |
| ***Presumed healthy dietary patterns***^e^ |  |  |  |
| **AHEI-2010** |  |  |  |
| Age- and energy-adjusted^a^ | 1 (reference) | 1.16 (0.85-1.59) | 1.19 (0.87-1.61) |
| Multivariable-adjusted^b^ | 1 (reference) | 1.18 (0.86-1.62) | 1.25 (0.91-1.70) |
| **aMED** |  |  |  |
| Age- and energy-adjusted^a^ | 1 (reference) | 0.89 (0.66-1.21) | 0.89 (0.65-1.23) |
| Multivariable-adjusted^b^ | 1 (reference) | 0.91 (0.67-1.23) | 0.93 (0.68-1.29) |
| **DASH** |  |  |  |
| Age- and energy-adjusted^a^ | 1 (reference) | 1.20 (0.88-1.63) | 0.89 (0.65-1.23) |
| Multivariable-adjusted^b^ | 1 (reference) | 1.22 (0.89-1.65) | 0.93 (0.67-1.28) |
| **Prudent** |  |  |  |
| Age- and energy-adjusted^a^ | 1 (reference) | 1.09 (0.79-1.50) | 1.09 (0.78-1.52) |
| Multivariable-adjusted^b^ | 1 (reference) | 1.09 (0.80-1.50) | 1.11 (0.79-1.55) |
| ***Presumed unhealthy dietary patterns*^e^** |  |  |  |
| **Western** |  |  |  |
| Age- and energy-adjusted^a^ | 1 (reference) | 1.15 (0.85-1.57) | 1.09 (0.75-1.58) |
| Multivariable-adjusted^b^ | 1 (reference) | 1.11 (0.82-1.51) | 1.02 (0.70-1.49) |
| **EDIP** |  |  |  |
| Age- and energy-adjusted^a^ | 1 (reference) | 1.22 (0.89-1.67) | 1.43 (1.05-1.95) |
| Multivariable-adjusted^b^ | 1 (reference) | 1.21 (0.88-1.67) | 1.39 (1.02-1.89) |
| **EDIR** |  |  |  |
| Age- and energy-adjusted^a^ | 1 (reference) | 1.15 (0.85-1.56) | 1.08 (0.79-1.48) |
| Multivariable-adjusted^b^ | 1 (reference) | 1.14 (0.84-1.54) | 1.04 (0.75-1.43) |
| **EDIH** |  |  |  |
| Age- and energy-adjusted^a^ | 1 (reference) | 1.21 (0.89-1.64) | 1.41 (1.01-1.98) |
| Multivariable-adjusted^b^ | 1 (reference) | 1.18 (0.86-1.60) | 1.33 (0.95-1.87) |
| Abbreviation: HR, hazard ratio; CI, confidence interval; SD, standard deviation; AHEI, alternate healthy eating index-2010; aMED, alternate Mediterranean diet; DASH, dietary approaches to stop hypertension; EDIP, empirical dietary inflammatory pattern; EDIR, empirical dietary index for insulin resistance; EDIH, empirical dietary index for hyperinsulinemia; BMI, body mass index.  ^a^ Adjusted for age in years and cumulative average energy intake (continuous).  ^b^ Additionally adjusted for cumulative average BMI (continuous).  ^c^ Sex-specific standard deviation was used.  ^d^ Cases/person-years: 215/1,709,737 for women and 263/1,082,520 for men.  ^e^ For the presumed healthy dietary patterns in the top half of the table, the reference group includes participants with a dietary pattern score in the lowest tertile (e.g., least adherence to the healthier dietary pattern). For the presumed unhealthy dietary patterns in the lower half of the table, the reference group includes participants with a dietary pattern score in the lowest tertile (e.g. least adherence to the less healthy dietary pattern). | | | |

| **Table S9. Association between cumulative average dietary pattern and multiple myelomaⱡ risk (pooled results of women and men)** | |
| --- | --- |
|  | **HR (95% CI) per 1-SD increase^c^** |
| **AHEI-2010** |  |
| Age- and energy-adjusted^a^ | 1.03 (0.94-1.13) |
| Multivariable-adjusted^b^ | 1.05 (0.95-1.15) |
| **aMED** |  |
| Age- and energy-adjusted^a^ | 0.95 (0.86-1.05) |
| Multivariable-adjusted^b^ | 0.97 (0.87-1.07) |
| **DASH** |  |
| Age- and energy-adjusted^a^ | 0.96 (0.87-1.05) |
| Multivariable-adjusted^b^ | 0.97 (0.88-1.07) |
| **Prudent** |  |
| Age- and energy-adjusted^a^ | 0.95 (0.85-1.05) |
| Multivariable-adjusted^b^ | 0.95 (0.86-1.06) |
| **Western** |  |
| Age- and energy-adjusted^a^ | 1.05 (0.92-1.19) |
| Multivariable-adjusted^b^ | 1.01 (0.89-1.15) |
| **EDIP** |  |
| Age- and energy-adjusted^a^ | 1.12 (1.02-1.24) |
| Multivariable-adjusted^b^ | 1.09 (0.99-1.21) |
| **EDIR** |  |
| Age- and energy-adjusted^a^ | 1.06 (0.96-1.17) |
| Multivariable-adjusted^b^ | 1.03 (0.93-1.14) |
| **EDIH** |  |
| Age- and energy-adjusted^a^ | 1.11 (1.00-1.24) |
| Multivariable-adjusted^b^ | 1.07 (0.96-1.20) |
| Abbreviation: HR, hazard ratio; CI, confidence interval; SD, standard deviation; AHEI-2010, alternate healthy eating index-2010; aMED, alternate Mediterranean diet; DASH, dietary approaches to stop hypertension; EDIP, empirical dietary inflammatory pattern; EDIR, empirical dietary index for insulin resistance; EDIH, empirical dietary index for hyperinsulinemia; BMI, body mass index.  ^a^ Adjusted for age in years and cumulative average energy intake (continuous).  ^b^ Additionally adjusted for cumulative average BMI (continuous).  ^c^ Sex-specific standard deviation was used. | |

| **Table S10. Association of cross-classified cumulative average dietary pattern and BMI with multiple myeloma risk (pooled results of women and men)** | | | |
| --- | --- | --- | --- |
|  | **Healthiest** | **Medium** | **Unhealthiest** |
| ***Presumed healthy***  ***dietary patterns*** ^a^ | **Tertile 3** | **Tertile 2** | **Tertile 1** |
| **AHEI-2010** |  |  |  |
| BMI<25 |  |  |  |
| Cases | 73 | 74 | 49 |
| HR (95% CI) | 1 (reference)^a^ | 1.27 (0.92-1.76) | 0.97 (0.67-1.40) |
| BMI≥25 |  |  |  |
| Cases | 99 | 95 | 88 |
| HR (95% CI) | 1.47 (1.08-1.99) | 1.27 (0.93-1.73) | 1.21 (0.88-1.66) |
| **aMED** |  |  |  |
| BMI<25 |  |  |  |
| Cases | 69 | 70 | 57 |
| HR (95% CI) | 1 (reference)^a^ | 1.10 (0.79-1.54) | 1.12 (0.78-1.61) |
| BMI≥25 |  |  |  |
| Cases | 92 | 95 | 95 |
| HR (95% CI) | 1.34 (0.98-1.84) | 1.24 (0.90-1.70) | 1.32 (0.96-1.82) |
| **DASH** |  |  |  |
| BMI<25 |  |  |  |
| Cases | 69 | 75 | 52 |
| HR (95% CI) | 1 (reference)^a^ | 1.43 (1.03-2.00) | 1.18 (0.82-1.70) |
| BMI≥25 |  |  |  |
| Cases | 96 | 97 | 89 |
| HR (95% CI) | 1.47 (1.07-2.00) | 1.41 (1.03-1.93) | 1.44 (1.04-1.99) |
| **Prudent** |  |  |  |
| BMI<25 |  |  |  |
| Cases | 69 | 66 | 61 |
| HR (95% CI) | 1 (reference)^a^ | 1.09 (0.77-1.54) | 1.11 (0.77-1.59) |
| BMI≥25 |  |  |  |
| Cases | 103 | 93 | 86 |
| HR (95% CI) | 1.35 (0.99-1.84) | 1.24 (0.90-1.71) | 1.28 (0.91-1.79) |
| ***Presumed unhealthy dietary patterns ^a^*** | **Tertile 1** | **Tertile 2** | **Tertile 3** |
| **Western** |  |  |  |
| BMI<25 |  |  |  |
| Cases | 75 | 66 | 55 |
| HR (95% CI) | 1 (reference)^a^ | 1.12 (0.80-1.57) | 1.10 (0.75-1.62) |
| BMI≥25 |  |  |  |
| Cases | 81 | 112 | 89 |
| HR (95% CI) | 1.17 (0.85-1.61) | 1.50 (1.11-2.03) | 1.20 (0.85-1.70) |
| **EDIP** |  |  |  |
| BMI<25 |  |  |  |
| Cases | 52 | 77 | 67 |
| HR (95% CI) | 1 (reference)^a^ | 1.55 (1.08-2.20) | 1.78 (1.23-2.56) |
| BMI≥25 |  |  |  |
| Cases | 82 | 86 | 114 |
| HR (95% CI) | 1.72 (1.21-2.45) | 1.56 (1.10-2.21) | 1.82 (1.31-2.54) |
| **EDIR** |  |  |  |
| BMI<25 |  |  |  |
| Cases | 67 | 80 | 49 |
| HR (95% CI) | 1 (reference)^a^ | 1.29 (0.93-1.80) | 1.06 (0.73-1.55) |
| BMI≥25 |  |  |  |
| Cases | 84 | 93 | 105 |
| HR (95% CI) | 1.40 (1.01-1.94) | 1.38 (1.00-1.89) | 1.32 (0.96-1.81) |
| **EDIH** |  |  |  |
| BMI<25 |  |  |  |
| Cases | 66 | 70 | 60 |
| HR (95% CI) | 1 (reference)^a^ | 1.41 (1.00-1.98) | 1.70 (1.18-2.47) |
| BMI≥25 |  |  |  |
| Cases | 86 | 98 | 98 |
| HR (95% CI) | 1.61 (1.16-2.22) | 1.56 (1.14-2.15) | 1.57 (1.13-2.19) |
| Abbreviation: HR, hazard ratio; CI, confidence interval; AHEI-2010, alternate healthy eating index-2010; aMED, alternate Mediterranean diet; DASH, dietary approaches to stop hypertension; EDIP, empirical dietary inflammatory pattern; EDIR, empirical dietary index for insulin resistance; EDIH, empirical dietary index for hyperinsulinemia; BMI, body mass index.  All models adjusted for age in years and cumulative average energy intake (continuous).  ^a^ Reference group is those with low BMI and the healthiest dietary pattern. For the presumed healthy dietary patterns in the top half of the table, the reference group includes participants with a low BMI and a dietary pattern score in the highest tertile (e.g., greatest adherence to the healthy dietary pattern), whereas the highest-risk group includes participants with a higher BMI and a dietary pattern score in the lowest tertile (e.g., least adherence to the healthier dietary pattern). For the presumed unhealthy dietary patterns in the lower half of the table, the reference group includes participants with a low BMI and a dietary pattern score in the lowest tertile (e.g. least adherence to the less healthy dietary pattern), whereas the highest-risk group includes participants with a higher BMI and a dietary pattern score in the highest tertile (e.g., greatest adherence to the less healthy dietary pattern). | | | |

| **Table S11. Association between cumulative average dietary pattern and multiple myeloma risk stratified by BMI (pooled results of women and men)** | | |
| --- | --- | --- |
|  | **HR (95% CI) per 1-SD increase^a^** | **P for interaction^b^** |
| **AHEI-2010** |  |  |
| BMI<25 | 0.99 (0.86-1.15) | 0.27 |
| BMI≥25 | 1.09 (0.96-1.24) |  |
| **aMED** |  |  |
| BMI<25 | 0.90 (0.77-1.05) | 0.07 |
| BMI≥25 | 1.03 (0.89-1.18) |  |
| **DASH** |  |  |
| BMI<25 | 0.91 (0.78-1.06) | 0.26 |
| BMI≥25 | 1.00 (0.88-1.15) |  |
| **Prudent** |  |  |
| BMI<25 | 0.91 (0.77-1.08) | 0.28 |
| BMI≥25 | 0.98 (0.85-1.12) |  |
| **Western** |  |  |
| BMI<25 | 1.18 (0.96-1.43) | 0.46 |
| BMI≥25 | 0.92 (0.78-1.09) |  |
| **EDIP** |  |  |
| BMI<25 | 1.29 (1.10-1.52) | 0.03 |
| BMI≥25 | 0.99 (0.87-1.12) |  |
| **EDIR** |  |  |
| BMI<25 | 1.18 (1.00-1.40) | 0.64 |
| BMI≥25 | 0.96 (0.84-1.09) |  |
| **EDIH** |  |  |
| BMI<25 | 1.27 (1.07-1.50) | 0.30 |
| BMI≥25 | 0.96 (0.83-1.11) |  |
| Abbreviation: HR, hazard ratio; CI, confidence interval; SD, standard deviation; AHEI-2010, alternate healthy eating index-2010; aMED, alternate Mediterranean diet; DASH, dietary approaches to stop hypertension; EDIP, empirical dietary inflammatory pattern; EDIR, empirical dietary index for insulin resistance; EDIH, empirical dietary index for hyperinsulinemia; BMI, body mass index.  All models adjusted for age in years, cumulative average energy intake (continuous) and cumulative average BMI (continuous) (BMI was further adjusted within the strata).  ^a^ Sex-specific standard deviation was used.  ^b^ P for interaction between each dietary pattern (continuous) and BMI (binary). | | |
